# Metataxonomics reveal vultures as a reservoir for *Clostridium perfringens*

**DOI:** 10.1038/emi.2016.137

**Published:** 2017-02-22

**Authors:** Xiangli Meng, Shan Lu, Jing Yang, Dong Jin, Xiaohong Wang, Xiangning Bai, Yumeng Wen, Yiting Wang, Lina Niu, Changyun Ye, Ramon Rosselló-Móra, Jianguo Xu

**Affiliations:** 1 Department of New Pathogen, State Key Laboratory for Infectious Disease Prevention and Control, National Institute for Communicable Disease Control and Prevention, Chinese Center for Disease Control and Prevention, Beijing 102206, China; 2 Collaborative Innovation Center for Diagnosis and Treatment of Infectious Diseases, Hangzhou 310003, China; 3 Marine Microbiology Group, Department of Ecology and Marine Resources, Mediterranean Institute for Advanced Studies [Spanish National Research Council (CSIC)-University of the Balearic Islands (UIB)], E-07190 Esporles, Balearic Islands 07190, Spain

**Keywords:** *Clostridium perfringens*, full-length 16S rRNA gene, metataxonomics, operational phylogenetic unit, PacBio, vulture

## Abstract

The Old World vulture may carry and spread pathogens for emerging infections since they feed on the carcasses of dead animals and participate in the sky burials of humans, some of whom have died from communicable diseases. Therefore, we studied the precise fecal microbiome of the Old World vulture with metataxonomics, integrating the high-throughput sequencing of almost full-length small subunit ribosomal RNA (16S rRNA) gene amplicons in tandem with the operational phylogenetic unit (OPU) analysis strategy. Nine vultures of three species were sampled using rectal swabs on the Qinghai-Tibet Plateau, China. Using the Pacific Biosciences sequencing platform, we obtained 54 135 high-quality reads of 16S rRNA amplicons with an average of 1442±6.9 bp in length and 6015±1058 reads per vulture. Those sequences were classified into 314 OPUs, including 102 known species, 50 yet to be described species and 161 unknown new lineages of uncultured representatives. Forty-five species have been reported to be responsible for human outbreaks or infections, and 23 yet to be described species belong to genera that include pathogenic species. Only six species were common to all vultures. *Clostridium perfringens* was the most abundant and present in all vultures, accounting for 30.8% of the total reads. Therefore, using the new technology, we found that vultures are an important reservoir for *C*. *perfringens* as evidenced by the isolation of 107 strains encoding for virulence genes, representing 45 sequence types. Our study suggests that the soil-related *C. perfringens* and other pathogens could have a reservoir in vultures and other animals.

## INTRODUCTION

Animal-associated microbes have received immense attention because of their relevance to human health.^1^ The majority of human pathogens are of animal origin and especially have wild animals as host reservoirs.^2, 3^ Studying the microbiome of wild animals will provide valuable information to deliver an advance alert of or prevent putative infection outbreaks. Vultures are nature's most successful scavengers, feeding on the carcasses of dead animals without suffering any apparent illness effect. They perform an important task in removing rotting carcasses that could otherwise be a source of disease. However, they may also constitute a reservoir of pathogens that could be widely disseminated. Sky burial is practiced on the Qinghai-Tibet Plateau, and in Bhutan, Nepal and some parts of India.^4^ Old World vultures are attracted to the ritual and play a major role in removing the carcasses from the ritual sites. Our driving hypothesis was that vultures could acquire and disseminate pathogens from dead animals, such as *Marmota himalayana*, a major reservoir of *Yersinia pestis* on the Qinghai-Tibet Plateau.^5^

16S rRNA sequencing is the most widely used technique for microbial identification and diversity analysis. Metataxonomics is defined as the use of high-throughput 16S rRNA amplicon sequencing along with phylogenetic analysis to characterize the entire microbiota of a given sample.^6^ The major problem with most of the previous studies is that they rely on the use of platforms, such as Illumina or Roche 454, that generate only partial 16S rRNA gene sequences, mostly from hypervariable regions of no more than 600 bp in length and in general <400 bp. The PacBio platform offers the possibility to sequence almost full-length 16S rRNA gene amplicons, increasing the information content by at least 3 ×, which is necessary for an accurate phylogenetic inference.^7^ Despite some restrictions of PacBio caused by its higher error rate compared with other platforms,^8^ our approach minimizes their effect, as it does not rely on just clustering sequences at a given identity threshold in operational taxonomic units (OTUs). We use the operational phylogenetic unit (OPU) strategy that is based on phylogenetic inference after a treeing approach, which, among other benefits, diminishes the influence of sequence errors and indels.^9, 10^ The additional advantage of using almost full-length amplicon sequences is that the phylogenetic inference can be done after a *de novo* tree reconstruction instead of just the addition of partial sequences using the parsimony algorithm.^7^ This is probably the most accurate way to analyze the diversity based on 16S rRNA gene amplicons.

## MATERIALS AND METHODS

### Vulture sampling

The vultures were live-captured in Qinghai Province, China. All animals were anesthetized, sampled using rectal swabs and released. The species of vultures were identified by mitochondrial cytochrome *c* oxidase I or *b* sequence analysis, amplified from the feathers.^11, 12^ The animal welfare practices associated with this study were approved by the Wildlife Protection Agents of Qinghai Province.

### High-throughput 16S rRNA amplicon sequencing by PacBio and Illumina MiSeq systems

Genomic DNA was extracted from rectal swabs using the QIAamp Fast DNA Stool Kit (Qiagen, Hilden, Germany). For the PacBio sequencing, the almost complete region of the bacterial 16S rRNA gene was amplified using the universal primer set 27F (5′-AGA GTT TGA TCC TGG CTC AG-3′) and 1492R (5′-GNT ACC TTG TTA CGA CTT-3′).^13^ PCR was performed using *Ex Taq* DNA polymerase, and the parameters for amplification were as follows: initial denaturation for 5 min at 94 °C; 25 cycles of denaturation at 94 °C for 30 s, annealing at 52 °C for 30 s, and extension at 72 °C for 1 min 30 s; and finally an extension step at 72 °C for 7 min. Sequencing was conducted on a PacBio RS II platform at TianJin Biochip Corporation, China. The primary 16S rRNA sequences generated were filtered using the SMRT Portal (version 2.3.0; www.pacb.com/devnet/). To ensure that the barcoded reads are correctly assigned to their original samples, a minimum barcode score of 22 was selected to achieve 99.5% accuracy. Data containing ambiguous bases was removed, primer sequences and adapters were excised from the filtered reads, and sequences outside of the 10–1490 nucleotide positions were trimmed.

For the Illumina MiSeq sequencing, the PCR amplification of the V3-V4 region of the bacterial 16S rRNA was performed using universal primers 341F (5′-CCT ACG GGN GGC WGC AG-3′) and 805R (5′-GAC TAC HVG GGT ATC TAA TCC-3′).^14^ The cycling parameters for amplifying V3-V4 using *Ex Taq* DNA Polymerase were as follows: initial denaturation for 5 min at 94 °C; 25 cycles of denaturation at 94 °C for 30 s, annealing at 55 °C for 30 s and elongation at 72 °C for 30 s; and a final step at 72 °C for 7 min. Sequencing was conducted using a paired-end 2 × 300 bp cycle run on an Illumina MiSeq platform at Berry Genomics Co., Ltd, Beijing, China. The image analysis, base calling and error estimation were performed using Illumina Real-Time Analysis software. The paired-end reads produced by Illumina Miseq were merged using FLASH.^15^

Qiime was applied for further filtering.^16^ Chimeras were removed using the UCHIME algorithm and using RDP gold as a reference database.^17, 18^ Rarefaction curves using PAST^19^ software, version 1.82b, and Good's coverage were calculated as previously indicated.^9^ The microbiome raw sequence data of this study have been deposited in the NCBI Sequence Read Archive (SRA) with the accession code SRP082183.

### Operational taxonomic unit and operational phylogenetic unit analysis strategy

The USEARCH pipeline was used to cluster the 16S rRNA sequences of both sequencing platforms into OTUs with a threshold set at 98.7% identity.^20^ The most frequent read of each OTU was selected as a representative to be added to the LTP123 database (The All-Species Living Tree Project)^21^ and aligned using the SINA tool (SILVA Incremental Aligner).^22^ The aligned sequences were inserted into the default tree using the Parsimony tool implemented in the ARB software package.^23^ The resulting insertions were manually inspected to recognize all representative sequences affiliating closely with either type strain sequences or clearly within a genus lineage. All sequences that remained unaffiliated were added to the SILVA SSURef_NR_123 (Silva Reference Non Redundant) database and inserted into the default tree.^22^ Approximately three closest relative sequences representing uncultured organisms were selected for each independent lineage made by the OTU representatives and exported to the LTP123 database.

A subset of sequences containing (i) all PacBio OTU representative sequences, (ii) the selection of the reference type strains and the SILVA REF123 recruited sequences, and (iii) the neighbor-joining supporting sequence data set was used to reconstruct a neighbor-joining tree.^24^ The reconstruction was performed using a 30% conservational filter to avoid phylogenetic noise. The partial Illumina MiSeq sequences obtained and the downloaded Roche 454 sequences of the New World vulture study^25^ were posteriorly added with the Parsimony tool to the final tree. The reconstruction was manually inspected, and each unique lineage including OTU representatives and at least one reference sequence was recognized as an OPU.^10, 26^ The sequences that were identical or nearly identical (>98.7% identity) to the type strain sequences were considered members of the same species. When the OPU represented an independent lineage within a genus, it was considered a new species of this taxon. Unique lineages affiliated with outside known genera were considered uncultured lineages of known families, orders or classes. The sequencing error rate was determined for each sample as previously decribed.^8^ Insertion and deletion rates were obtained by comparing the final alignment of all representative selected sequences with the corresponding reference sequence in the LTP123.

### Microbiome-guided culturing

The species with putative medical significance as suggested by the microbiome analysis, such as *C. perfringens*, were attempted to be isolated from fecal swabs and analyzed for virulence genes and genetic diversity.^27, 28, 29^

## RESULTS

### Use of the almost full-length 16S rRNA gene for OPU assignment

The PacBio platform rendered a total of 89 412 raw 16S rRNA sequence reads for the nine vultures, with an average of 9935±1182 each. After quality filtering and the removal of chimeras, a total of 54 135 (64.1%) high-quality reads were retained, with an average 6015±1058 per vulture, ranging from 3914 to 7501 reads. The sequences were on average 1442±6.9 bp in length. The sequences were clustered into 4223 OTUs at 98.7% identity, with an average of 469±257 OTUs per vulture (Table 1). The representative sequences for each OTU were used for the OPU design, in which an OPU is the smallest monophyletic group of sequences containing OTU representatives together with the closest reference sequence, including the sequence of a type strain when possible.^9^ The 4223 OTUs were classified into 314 OPUs, with 78±49.6 per vulture (Table 1). The amplification for the V3-V4 region using the Illumina MiSeq system rendered a total of 826 772 high-quality reads, with a mean of 91 864±23 335 per vulture, with average of 443±3.1 bp in length. Using a threshold of 98.7% identity, an average of 71±26.2 OTUs per vulture was obtained. The selected sequences of each OTU were affiliated with 126 OPUs, with an average of 31±14.4 per vulture (Table 1).

Trimming and quality control rendered similar percentage yields, with 62.6%±8.4% and 49.0%±13.8% for PacBio and Illumina, respectively (Supplementary Table S1). In accordance with the rarefaction analysis, the 314 OPUs detected by PacBio did not enter saturation, whereas for the 126 MiSeq OPUs, the curves were well-saturated (Supplementary Figure S1). The error rates generated by the PacBio sequencing were slightly higher than those of Illumina MiSeq (Supplementary Figure S1). Three of the four most abundant OPUs (OPU107, OPU123 and OPU236), gathering 57.9% of the total reads and summing to 1540 representative OTU sequences, were selected for a deeper analysis of the insertion and deletion rates compared with the corresponding type strain reference sequence in the LTP123.^21^ For PacBio (Supplementary Table S2), the insertion and deletion rates were 0.4%±0.96 and 2.37%±1.25, respectively, accounting for mean inserted and deleted nucleotides in each sequence of 5.9±14.3 and 33.3±17.2. For the Illumina MiSeq system, the insertion and deletion rates were 0.06%±0.23 and 0.1%±0.17, respectively, accounting for mean inserted and deleted nucleotides in each sequence of 0.29±1 and 0.44±0.77. As the phylogenetic inferences in each case were done using a 30% conservational filter, the influence of the insertions was negligible and that of the deletions was also low.

### Fecal microbiome profile at high taxon levels

**Figure 1:**
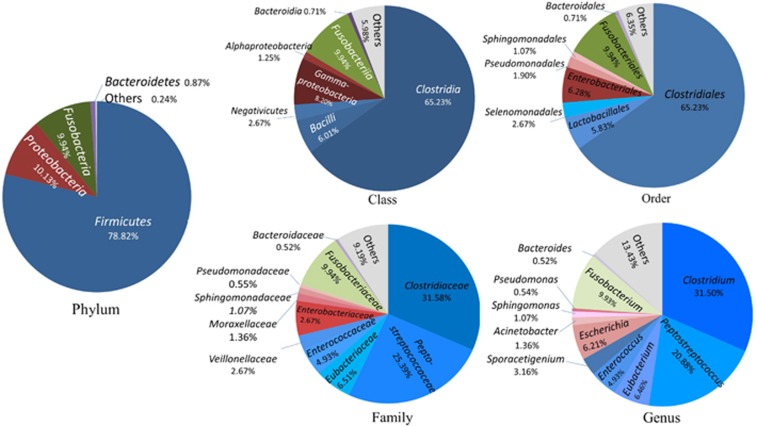
Microbiome profile at phylum, class, order, family and genus categories of the Old World vultures. Phylum: of 13 phyla classified, only four with >0.1% total reads are displayed. Class: of 25 classes classified, only seven with >0.5% total reads are displayed. Order: of 37 orders classified, only eight with>0.5% total reads are displayed. Family: of 66 families classified, only 11 with>0.5% total reads are displayed. Genus: of 89 genera classified, only 11 with >0.5% total reads are displayed.

The 314 OPUs obtained with the PacBio approach were affiliated with 13 phyla, 25 classes, 37 orders, 66 families and 89 genera (Figure 1). Only two of the 13 phyla were detected in all vultures, namely, *Firmicutes* and *Proteobacteria*. Four of the 25 classes were detected in all vultures, namely, *Clostridia, Gammaproteobacteria, Bacilli* and *Alphaproteobacteria.* Four of the 37 orders were detected in all vultures, namely, *Clostridiales*, *Enterobacteriales, Lactobacillales* and *Sphingomonadales.* Six of the 66 families were detected in all vultures, namely, *Clostridiaceae, Peptostreptococcaceae, Eubacteriaceae, Sphingomonadaceae, Peptoniphilaceae* and *Lactobacillaceae*. Seven of the 89 genera were detected in all vultures, namely, *Clostridium*, *Peptostreptococcus*, *Peptoniphilus*, *Eubacterium*, *Sporacetigenium*, *Sphingomonas* and *Lactobacillus* (Figure 1).

### Species composition of the fecal microbiomes

**Figure 2:**
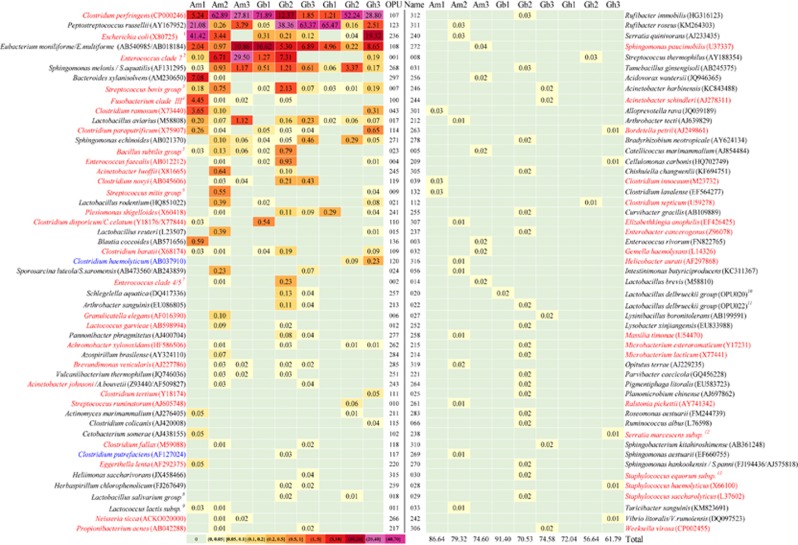
Abundance heat map of the 102 known species detected and their medical significance. The abundances are shown as percentage of reads in each vulture, and highlighted with a color scale. Species on the list of medically significant bacteria are highlighted in red. The species in blue were reported to be isolated from diseased animals. The species in black have no information to be associated with human or animal disease. The species *Gyps himalayensis*, *Gypaetus barbatus* and *Aegypius monachus* were abbreviated as Gh, Gb and Am. The number following the abbreviation was the number for a given sampled vulture. #: OPUs including several species (for example, ^1^OPU236, *E. coli* or *Shigella*).

Of the 314 OPUs detected, 102 (32%) were identified as members of known species, as they were very closely affiliated (>98.7% identity) or exhibited an identical sequence to their respective type strain (Figure 2). In some cases, due to the high identity between type strain sequences, an accurate identification was not possible, and the OPU was identified by the distinct members of the clade, such as OPU001, identified as *Enterococcus clade 7*, including several species;^30^ and OPU108 and OPU268, identified as *Eubacterium moniliforme/E. multiforme* and *Sphingomonas melonis/S. aquatilis*, respectively.

The 102 known species gathered between 57.6% and 91.4% of the reads in each vulture and 73.0% of the total reads for all vultures. The abundances of individual species in individual vultures varied significantly, ranging from 0.002% to 71.9% reads (Figure 2). Only four characterized species were present in all vultures, namely, *C. perfringens* (OPU107)*, Peptostreptococcus russellii* (OPU123), *E. moniliforme/E. multiforme* (OPU108), and *S. melonis/S. aquatilis* (OPU268). In addition, there were only five species with >1% total reads, i.e., *C. perfringens* (OPU107; 1.2%–71.9%), *P. russellii* (OPU123; 0.05%–65.5%), *Escherichia coli/Shigella* (OPU236; 0.04%–41.42%, but absent in Am3 and Gh1), *E. moniliforme/E. multiforme* (OPU108; 0.22%–16.6%) and the *Enterococcus clade 7* (0.1%–29.5% Figure 2; Supplementary Table S3).

Of the 102 known species, 45 were considered medically significant bacteria or had been reported to be responsible for outbreaks or infections in humans, as evidenced by literature searches for the combination of the given species name and outbreak^31^ (Figure 2), and ranged from three to 25 in the individual vultures. The analyses evidenced outbreak causative agents including *Plesiomonas shigelloides* (OPU241), *C. perfringens* (OPU107), *C. septicum* (OPU112), *C. tertium* (OPU111), *C. barati* (OPU109), *Bordetella petrii* (OPU263), *Brevundimonas vesicularis* (OPU285), *Gemella haemolysans* (OPU032), *Granulicatella elegans* (OPU006), *Propionibacterium acnes* (OPU217), *Serratia marcescens* (OPU238), *Ralstonia pickettii* (OPU261), *Sphingomonas paucimobilis* (OPU272), *Staphylococcus haemolyticus* (OPU028), and *S. saccharolyticus* (OPU029), among others.^31^

**Figure 3:**
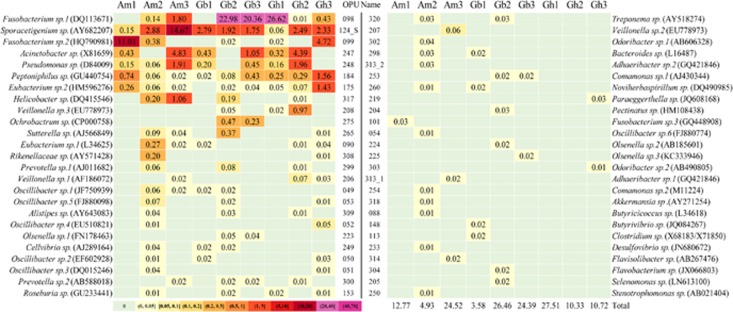
Abundance heatmap of the 50 yet to be described bacterial species.

Of the 314 OPUs, 50 affiliated with 34 known genera (Figure 3) but represented independent lineages with identity values <98.7% from any type strain within each respective genus as an indication that they may represent new unclassified species. Therefore, we considered them yet to be described species. These 50 new species accounted for between 3.6% and 27.5% of the reads in the individual vultures and 15.9% of the total reads for all vultures (Figure 3; Supplementary Table S4). Only *Sporacetigenium sp*. (OPU124_S) and *Peptoniphilus sp*. (OPU184) were detected in all nine vultures. Of the 34 genera with putative new species, 23 have species of pathogens that caused human infections or have been isolated from clinical specimens, such as *Acinetobacter* (OPU247)*, Alistipes* (OPU309)*, Bacteroides* (OPU298)*, Butyrivibrio* (OPU148)*, Clostridium* (OPU113)*, Comamonas* (OPU253)*, Desulfovibrio* (OPU233)*, Eubacterium* (OPU090)*, Fusobacterium* (OPU098)*, Helicobacter* (OPU317)*, Ochrobactrum* (OPU275)*, Odoribacter* (OPU302)*, Olsenella* (OPU223)*, Oscillibacter* (OPU049)*, Paraeggerthella* (OPU219)*, Peptoniphilus* (OPU184)*, Prevotella* (OPU299)*, Pseudomonas* (OPU248)*, Selenomonas* (OPU205)*, Stenotrophomonas* (OPU250)*, Sutterella* (OPU265)*, Treponema* (OPU320)*, Veillonella* (OPU206).

Of the 314 OPUs, 161 were considered unknown new lineages of uncultured representatives, which represented new branches affiliating outside any known genus but representing putative unclassified new lineages different from the known genera. These summed to between 0.4% and 33.0% of the reads of each individual vulture (Supplementary Table S5) and 11.0% of the total reads for all vultures. However, none of those unknown new lineages of uncultured representatives was detected in all nine vultures studied. In addition, OPU287 was identified as a chloroplast.

### Top 10 most abundant species and two-component model

**Figure 4:**
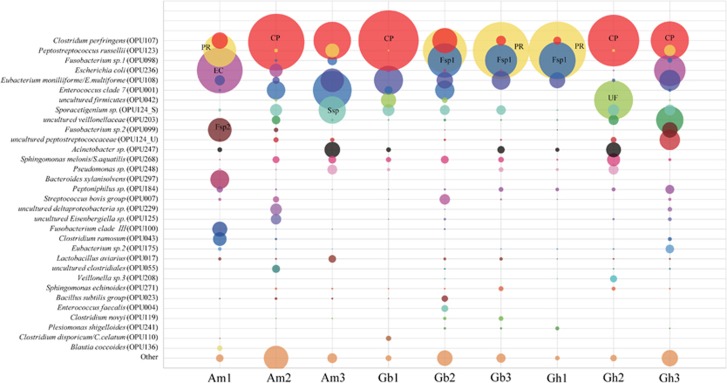
Abundances of the top ten most abundant OPUs. The abundances are shown as percentages of reads in each vulture. A total of 32 OPUs were collected as being the top 10 within the set of all vultures. The others indicated the rest of the OPUs for a given vulture.

The top ten most abundant OPUs of each vulture summed to a total of 32 OPUs, covering between 88.1% and 99.3% of their respective total reads (Figure 4, Supplementary Tables S3–S5). Only six OPUs were shared by all vultures, namely, *C. perfringens* (OPU107)*, P. russellii* (OPU123), *E. moniliiforme/E. multiforme* (OPU108)*, Sporacetigenium sp.* (OPU124_S)*, S. melonis/S. aquatilis* (OPU268) and *Peptoniphilus sp.* (OPU184). It was remarkable that the two OPUs (C+P) *C. perfringens* (OPU107) and *P. russellii* (OPU123) accounted for 51.7% of the total reads, ranging from 26.3% to 71.9% in each individual sample. The OPUs with total reads higher than 1% also included *Fusobacterium sp.* 1 (OPU098), *E. coli/Shigella* (OPU236), *Eubacterium moniliiforme/E. multiforme* (OPU_108), *Enterococcus clade 7* (OPU001), uncultured *Firmicutes* (OPU042), *Sporacetigenium sp*. (OPU124_S), uncultured *Veillonellaceae* (OPU203), *Fusobacterium sp*. 2 (OPU099), uncultured *Peptostreptococcaceae* (OPU124_U) and *Acinetobacter sp*. (OPU247) (Figure 4).

### Comparison between the PacBio and Illumina MiSeq approaches

Using the Illumina MiSeq platform, we detected a total of 126 OPUs of which only 44 (35%) were identified as known species, representing 43.9% of the total reads (Supplementary Tables S3–S6). The combination of the two approaches summed a total of 355 OPUs. Of these, the majority (87.5% or 83.1% of the total reads, respectively; Supplementary Figure S2) affiliated with OPUs common to the two approaches. Actually, the two methods produced equivalent relative abundances for all shared taxa. Only OPU123 (*P. russellii*) was underrepresented in the Illumina approach, a fact that is most likely related to amplification biases. Of the 355 OPUs, 229 (64.5%) were only detected by PacBio, accounting for 12.5% of the total reads. Finally, 41 OPUs were only detected by Illumina MiSeq, accounting for 16.8% of its total reads (Supplementary Figure S2, Supplementary Table S6).

### Microbiome profiles between Old World and New World vultures

We compared our data with those generated for the New World vultures.^25^ The 466 824 sequences of 435 bp in length affiliated with 105 OPUs (Supplementary Figure S3). The Old and New World vultures shared 40 OPUs, which accounted for 66.7 and 85.3% of the total reads, respectively. Only 65 OPUs were detected in the New World vultures (Supplementary Figure S3). The five most abundant OPUs shared by all vultures were *C. perfringens* (OPU107)*, P. russellii* (OPU123), *E. coli/Shigella* (OPU236), *Sporacetigenium sp.* (OPU124_S), and *Fusobacterium sp*. 2 (OPU099). The five most abundant species only detected in Old World vultures were *Fusobacterium sp.* 1 (OPU098), *E. moniliiforme/E. multiforme* (OPU108)*, Enterococcus clade 7* (OPU001), uncultured *Firmicutes* (OPU042) and uncultured *Veillonellaceae* (OPU203) (Supplementary Figure S3).

### Microbiome-guided culturing of *C. perfringens*

The species-level microbiome analysis indicated that the deadly pathogen *C. perfringens* was the most abundant commensal member in the intestinal flora of all vultures. Therefore, the specific isolation of the members of this species was conducted. A total of 107 strains of *C. perfringens w*ere isolated from the rectal swabs of the nine vultures (Supplementary Figure S4). All strains were identified by full-length 16S rRNA sequence analysis and tested positive for the major lethal alpha-toxin gene by the PCR method, which was then confirmed by sequencing. The toxin gene beta2 toxin gene (*cpb*2) was found in fifteen isolates belonging to two sequence types (STs), while other toxin genes were tested negative. The multi-locus sequence typing (MLST) approach based on eight genes grouped the 107 strains into 45 sequence types, including alpha-toxin gene (*plc*), D-alanine-D-alanine ligase gene (*ddlA*), deoxyuridine triphosphatase gene (*dut*), glycerol kinase gene (*glpK*), deoxyguanylate kinase gene (*gmk*), recombinase gene (*recA*), superoxide dismutase gene (*sod*) and triose phosphate isomerase gene (*tpiA*) (Supplementary Table S7).^27^ Twenty-seven STs contained a single strain. There were three predominant STs, namely, ST33, ST41, and ST45, containing 18, 11 and six strains, respectively. The neighbor-joining reconstruction based on the concatenated MLST sequences showed that the strains from the vultures were distributed into four groups, different from the avian isolates.

## DISCUSSION

Metataxonomic analysis with almost full-length 16S rRNA gene sequencing generated by the PacBio system allowed the precise identification of the microbiome composition up to the species category.^9, 10, 26^ Our study evidenced the benefits of using this approach as it (i) produces an almost complete gene sequence of the amplicons and (ii) seems to render higher-diversity yields of especially the low-abundance microbiota in comparison with the Illumina MiSeq approach. The fact that the rates of insertion and deletion were low, together with the capabilities to use general filters to remove phylogenetic noise^23^ and the *de novo* reconstruction based on phylogenetic inferences, provide the OPU recognition and identification with high confidence. The combination of PacBio and Illumina MiSeq data revealed the recognition of 355 OPUs that could be assumed to represent different individual species.^10^ The PacBio revealed approximately 65% and Illumina MiSeq approximately 12% OPUs that were not reciprocally detected. However, the coincident taxa detected represented >83% of the reads in both cases, and the relative proportions were generally equivalent. The Illumina MiSeq rendered much a lower resolution in revealing the low-abundance microorganisms, despite the number of sequences being approximately 15x higher. Using the metataxonomic approach, we could assign approximately 72.9% of the total reads to known species and 15.9% to yet to be described species. Actually, of the top 10 most abundant microbes in each individual vulture, approximately 72.2% of the total reads could be readily identified as members of known species and 15.3% as yet to be described species.

In this study, an especially remarkable finding was the unexpected abundance and predominance of *C. perfringens*, a well-known pathogen causing enteric diseases and gas gangrene.^32^ *C. perfringens* had been already detected as prevalent in healthy dogs by means of culture-dependent approaches.^33^ To compare our results with the New World vulture's microbiome,^25^ we applied the OPU approach to the available sequences and followed the same strategy as for other Roche 454 datasets with partial sequences.^9, 10^ Approximately 40 OPUs were shared by the two sample sets, with sequence contributions of >66% in the Old World and >85% in the New World. The shorter sequences, together with the OTU strategy used in the original study, restricted the accurate identification to only the genus level.^25^ However, we identified 88% as members of known species or new species within known genera. Within the shared microbiome, *C. perfringens* was again one of the most relevant species. Although an accurate species identification was not achieved, Roggenbuck already identified gene coding for the tissue-degrading enzymes and toxins of *C. perfringens*. The New World vultures seem to have a similar pattern of two components to that observed in our data set (C+P). In both cases, *C. perfringens* is one of the key players.^25^

The abundance of *C. perfringens* in all vultures yet studied brought us to believe that the relationships between *C. perfringens* and vultures could be mutualistic and not pathogenic. Therefore, both the New and Old World vultures in the USA and China seem to be important animal reservoirs for this pathogenic species. We also analyzed lineages of *C. perfringens* isolates associated with distinct disease presentations. These 107 isolates were identified into three evolutionary lineages: Lineage I, Gangrene, bovine hemorrhagic enteritis (β2+), antibiotic-associated diarrhea, retail meats (non-pathogenic); Lineage II, Human peritonitis and septicemia; and Lineage III, Bovine and equine hemorrhagic enteritis (β2-) (data not shown).^34^ An estimated 9.4 million cases of foodborne diseases occur each year in the US, and one million (10%) of them have been related to *C. perfringens* poisoning.^35^ Therefore, this finding has significance in public health, which was reinforced by the isolation of 107 strains from the nine vultures encoding for the virulence gene and the surprising diversity of STs and evolutionary lineages.

Most gastrointestinal infections by *C. perfringens* are caused by the contamination of food sources by humans or by animal feces. However, *C. perfringens* is ubiquitous in the soil and, thus, can be a source of gastrointestinal diseases^36, 37^ and gas gangrene, which was common in the combat injuries of soldiers well into the 20th century.^37^ Although we have no direct evidence of soilborne food poisoning or gas gangrene, our results indicate that the vulture's fecal deposits could be a means of dispersion of pathogenic bacteria, especially *C. perfringens* due to its high prevalence. In this regard, it was remarkable that both the *C. perfringens* isolates in this study and those recovered from the soil were predominantly type A.^36^

It seems that vultures could be an animal reservoir of many additional emerging bacterial pathogens, as from the 102 species identified in Old World vultures, 45 have been isolated from patients or were reported as responsible for outbreaks.^31^ It should be noted that both *R. pickettii* and *S. paucimobilis* have been recognized as emerging pathogens in hospital settings as causative agents for respiratory infections or meningitis. Very interestingly, both were isolated from soil samples.^38, 39, 40^ The results provide an almost complete inventory of the potentially zoonotic known bacteria in vultures.

In short, our metataxonomic approach offers a species-precise identification for microbiome profiling. It could be used for a pre-screening of putative pathogens for routine implementation in future large-scale epidemiological studies, for the culture-independent etiological investigation of infectious disease outbreaks, and as a culture-guidance methodology.

It should be noted that some of the Old World vultures on the Tibet-Qinghai Plateau are on the list of state-protected wildlife, including *Gypaetus barbatus*, *Gyps himalayensis* and *Aegypius monachus*. This wildlife protection regulation limited the number of vultures that could be sampled.

## Figures and Tables

**Table 1:** Quality, richness and diversity estimation of the 16S rRNA sequencing by PacBio and Illumina^a^

**Sample**	**PacBio**								**Illumina**							
	**Reads**	**Length (bp)**	**Q20 (%)**	**Q30 (%)**	**OTUs**	**OPUs**	**Shannon**	**Coverage**	**Reads**	**Length**	**Q20 (%)**	**Q30 (%)**	**OTUs**	**OPUs**	**Shannon**	**Coverage**
Am1	3914	1456±31.83	99.06	98.20	270	40	2.685	0.996	107 049	446±10.19	99.48	97.54	77	46	3.13	0.999999
Am2	6911	1438±29.82	99.03	98.18	1041	180	2.885	0.989	113 973	440±8.86	99.54	97.81	95	53	1.35	0.999997
Am3	5379	1445±31.49	99.02	98.16	380	52	2.761	0.994	43 454	440±11.29	97.55	91.28	16	12	1.72	0.999980
Gb1	5524	1433±28.74	99.08	98.25	321	46	1.459	0.995	109 183	442±8.07	99.48	97.57	68	21	1.58	0.999996
Gb2	6210	1437±30.25	99.19	98.46	518	128	2.977	0.990	107 243	444±8.47	99.51	97.62	108	40	2.00	1
Gb3	5613	1440±33.21	99.18	98.38	284	70	1.864	0.994	100 036	441±6.00	99.55	97.8	71	19	1.54	1
Gh1	6288	1437±30.77	99.05	98.22	346	30	1.368	0.999	98 151	441±5.03	99.55	97.83	79	17	1.64	0.999999
Gh2	6795	1446±32.17	99.16	98.33	341	51	2.054	0.997	73 172	448±11.31	99.52	97.6	51	33	1.93	0.999984
Gh3	7501	1445±29.77	99.07	98.23	722	101	3.288	0.994	74 511	447±9.92	99.44	97.42	72	39	2.98	0.999997
Average	6015±1057.91	1442±6.90	99.09	98.27	469±257.09	78±49.59	2.371	0.994	91 864±23 335.08	443±3.11	99.29	96.94	71±26.17	31±14.40	1.99	0.999995
Total	54 135				ND	314			826 772				ND	126		

Abbreviations: not done, ND; operational phylogenetic unit, OPU; operational taxonomic unit, OUT.

a The good coverage was calculated on OPUs.
