# Supplementary material for: Metataxonomics reveal vultures as a reservoir for Clostridium perfringens
Source: Emerg Microbes Infect. 2017 Feb 22;6(2):e9–. doi: 10.1038/emi.2016.137 (PMC5322324; doi:10.1038/emi.2016.137)
Supplement: Supplementary Figure 1 [file emi2016137x1.docx]

**Supplementary Figure S1** **Error rate and rarefaction curve for 16S rRNA sequences generated by PacBio and Illumina Miseq**

Supplementary Figure S1A**:** Error rate comparison of 16S rRNA sequences generated by PacBio or Illumina Miseq #

#: Error rate in each samples was calculated using qulality checked sequences. Error rate was calculated with follows formule. A base with quality score **q** implies the probability of being erroneously. The calculating relations between **q** and **P_q_** was shown in formula (1) and formula (2). Error rate (**errP**) of each samples was calculated with **q** of each base in a sample, as shown in formula (3), **n*_q_*** is the number of bases with quality score being **q**.


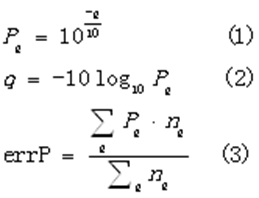


Supplementary Figure S1B**:** Rarefaction curves based on OTUs (right) and OPUs (left) for 16S rRNA sequences generated by Illumina Miseq

Supplementary Figure S1C**:** Rarefaction curves based on OTUs (right) and OPUs (left) for 16S rRNA sequences generated by PacBio
