# Supplementary figures and images for: Metataxonomics reveal vultures as a reservoir for Clostridium perfringens
Source: Emerg Microbes Infect. 2017 Feb 22;6(2):e9–. doi: 10.1038/emi.2016.137 (PMC5322324; doi:10.1038/emi.2016.137)

**Supplementary Figure S3 Comparative occurrence of OPUs of New and Old World vultures**


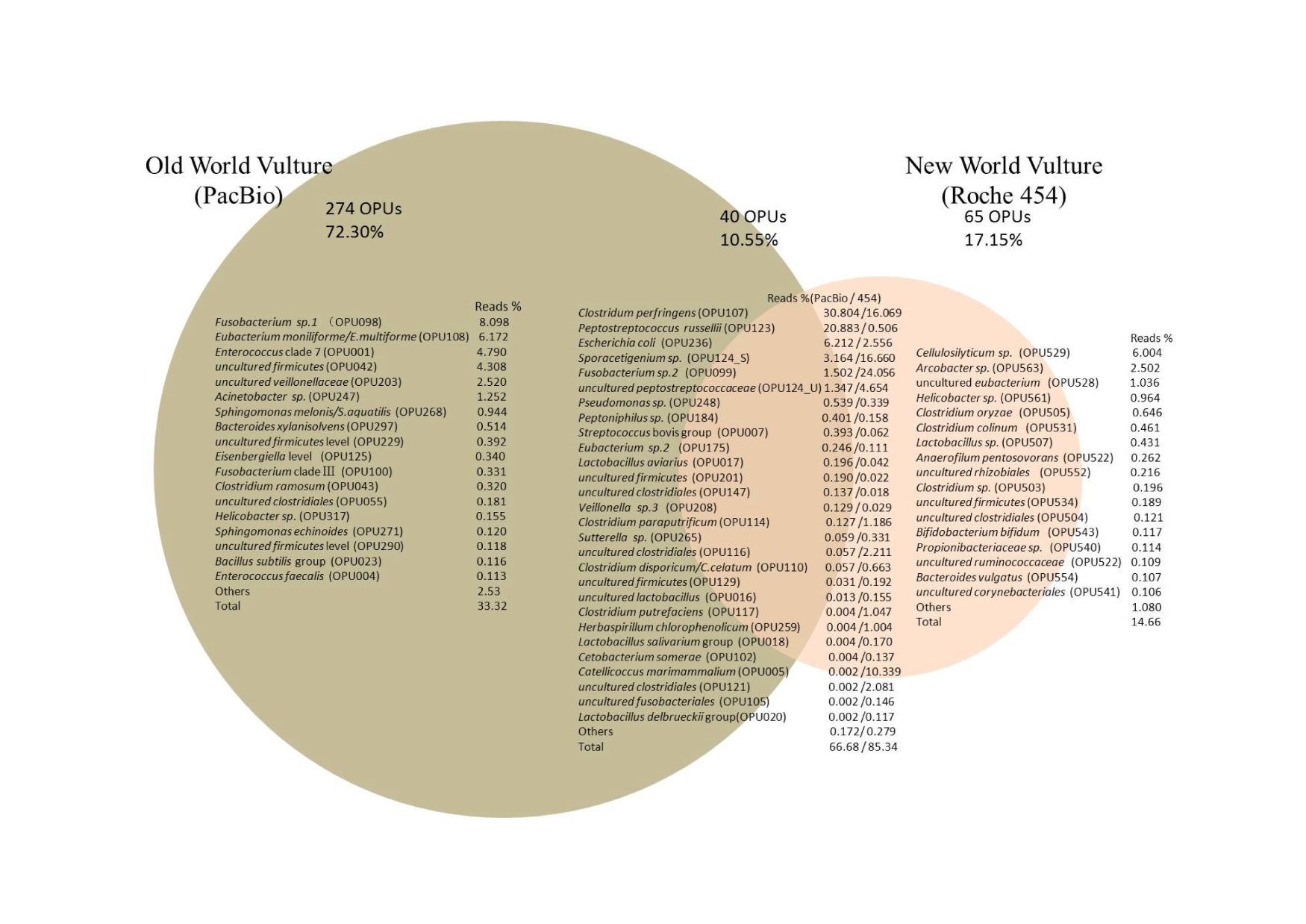

Supplement: Supplementary Figure 3 [file emi2016137x3.docx]
