# Supplementary material for: Metataxonomics reveal vultures as a reservoir for Clostridium perfringens
Source: Emerg Microbes Infect. 2017 Feb 22;6(2):e9–. doi: 10.1038/emi.2016.137 (PMC5322324; doi:10.1038/emi.2016.137)
Supplement: Supplementary Figure 4 [file emi2016137x4.docx]

**Supplementary Figure S4 Dendrogram of concatenated MLST sequences of *C. perfringens*** **isolated** **from old world vultures and from avian**.


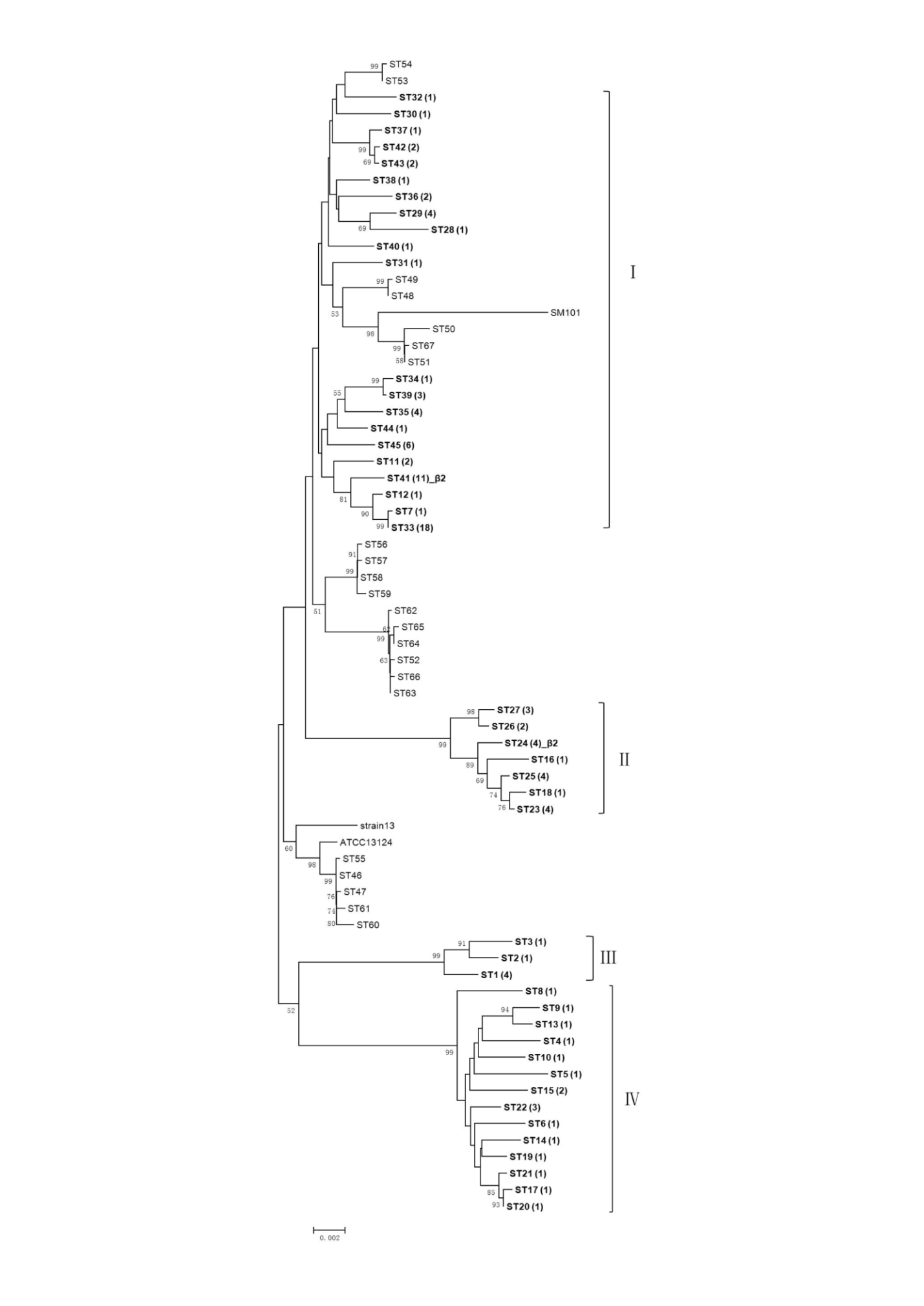


The sequences were analyzed using DNASTAR version7 software and phylogenetic analyses were conducted using the program MEGA 6 with the neighbor-joining method. The statistical reliability of internal branches was assessed from 1000 bootstrap analysis. The strains in bold were isolated in this study. ST46-ST67 were previously published for MLST analysis by Chalmers G. The number in brackets is the total isolates of each sequence type (ST). Three *C. perfringens* strains with bublished genome sequences (strain 13, canine isolate; SM101, food poisoning isolate; and ATCC 13124, human gas gangrene isolate). Toxin gene *cpb2* was found in fifteen isolates which were all belonged to two different STs (ST24 and ST41).  The 107 were classified into 45 sequence types STs (classified into 4 groups). Each sequence of these 45 ST found in this study has been uploaded to the GenBank database under the following accession number: KX711184-KX711228 for *plc*, the alpha toxin gene; KX711229-KX711273 for *ddlA*, the Dalanine-D-alanine ligase gene; KX711274-KX711318 for *dut*, the deoxyuridine triphosphatase gene; KX711319-KX711363 for *glpK*, the glycerol kinase gene; KX711364-KX711408 for *gmk*, the deoxyguanylate kinase gene; KX711409-KX711453 for *recA*, the recombinase gene; KX711454-KX711498 for *sod*, the superoxide dismutase gene and KX711499-KX711543 for *tpiA*, the triose phosphate isomerase gene.
