# Supplementary material for: Metataxonomics reveal vultures as a reservoir for Clostridium perfringens
Source: Emerg Microbes Infect. 2017 Feb 22;6(2):e9–. doi: 10.1038/emi.2016.137 (PMC5322324; doi:10.1038/emi.2016.137)
Supplement: Supplementary Table 1 [file emi2016137x5.docx]

**Supplementary Table S1 Quality control yields on the 16S rDNA sequencing for PacBio and illumina MiSeq**

|  | **PacBio** | | | | | | | | **Illumina MiSeq** | | | |
| --- | --- | --- | --- | --- | --- | --- | --- | --- | --- | --- | --- | --- |
| Animal | Raw reads | Non ambiguous | Primer  cutting | Length (1,200~1,400bp) | Qiime processed | Non  chimera | length  (≥1300bp) | Extract effective* | Raw reads | Combined | Qualified | Extract effective* |
| Am1 | 10 055 | 9 912 | 6 093 | 6 037 | 4 502 | 4 078 | 3 986 | 40.56 | 172 093 | 112 002 | 111 819 | 62.69 |
| Am2 | 11 145 | 10 959 | 9 099 | 8 995 | 7 349 | 7 238 | 7 134 | 64.94 | 197 028 | 134 168 | 134 059 | 59.04 |
| Am3 | 8 780 | 8 611 | 7 460 | 7 396 | 5 699 | 5 577 | 5 437 | 63.52 | 290 235 | 45 441 | 45 330 | 15.52 |
| Gb1 | 8 838 | 8 669 | 7 584 | 7 542 | 5 812 | 5 748 | 5 642 | 65.04 | 202 960 | 131 907 | 131 760 | 55.53 |
| Gb2 | 9 853 | 9 747 | 8 611 | 8 562 | 7 139 | 6 380 | 6 314 | 64.75 | 216 335 | 139 825 | 139 648 | 52.35 |
| Gb3 | 8 284 | 8 194 | 7 406 | 7 377 | 5 829 | 5 754 | 5 675 | 69.46 | 195 336 | 132 440 | 132 313 | 52.85 |
| Gh1 | 9 950 | 9 769 | 8 472 | 8 406 | 6 520 | 6 469 | 6 355 | 65.02 | 194 026 | 132 375 | 132 271 | 51.96 |
| Gh2 | 10 564 | 10 410 | 8 959 | 8 885 | 6 972 | 6 943 | 6 854 | 65.72 | 159 975 | 97 291 | 97 157 | 48.89 |
| Gh3 | 11 943 | 11 755 | 9 784 | 9 679 | 7 761 | 7 693 | 7 588 | 64.41 | 183 638 | 107 488 | 107 244 | 43.23 |
| **Average** | 9 935±1 181.97 | 9 781±1 163.10 | 8 163±1 124.77 | 8 098±1 106.98 | 6 398±1 025.07 | 6 209±1 075.69 | 6 109±1 075.92 | 62.60±8.43 | 201 292±37 278.88 | 114 771±29 775.39 | 114 622±29 771.98 | 49±13.78 |
| **Total** | 89 412 | 88 026 | 73 468 | 72 879 | 57 583 | 55 880 | 54 985 | NA# | 1 811 626 | 1 032 937 | 1 031 601 | NA# |

*:Extract effective is the proportion of high quality reads for analysis in the raw reads, and the number of high quality reads for analysis see to Table1.

#: NA, not applied.
