# Supplementary material for: Metataxonomics reveal vultures as a reservoir for Clostridium perfringens
Source: Emerg Microbes Infect. 2017 Feb 22;6(2):e9–. doi: 10.1038/emi.2016.137 (PMC5322324; doi:10.1038/emi.2016.137)
Supplement: Supplementary Table 2 [file emi2016137x6.docx]

**Supplementary Table S2 The rates of insertion and deletion of three OPUs from PacBio and Illumina platform**

|  | **Num.OTU** | **Reads %** | **Delet/Seq*** | **SD** | **Insert/Seq*** | **SD** | **Delet %** | **SD** | **Insert%** | **SD** |
| --- | --- | --- | --- | --- | --- | --- | --- | --- | --- | --- |
|  | **PacBio** | | | | | | | | | |
| OPU107 | 826 | 30.80 | 33.42 | 16.68 | 2.79 | 4.74 | 2.39 | 1.22 | 0.19 | 0.32 |
| OPU123 | 478 | 20.88 | 34.61 | 17.82 | 12.64 | 23.32 | 2.43 | 1.28 | 0.85 | 1.56 |
| OPU236 | 236 | 6.21 | 30.44 | 17.65 | 3.07 | 3.95 | 2.13 | 1.27 | 0.21 | 0.27 |
| All | 1 540 | 57.89 | 33.34 | 17.23 | 5.89 | 14.27 | 2.37 | 1.25 | 0.40 | 0.96 |
|  | **Illumina** | | | | | | | | | |
| OPU107 | 76 | 32.51 | 0.43 | 0.74 | 0.08 | 0.42 | 0.10 | 0.17 | 0.02 | 0.10 |
| OPU123 | 28 | 4.41 | 0.32 | 0.55 | 0.93 | 1.74 | 0.07 | 0.13 | 0.21 | 0.39 |
| OPU236 | 8 | 4.14 | 0.88 | 1.46 | 0.00 | 0.00 | 0.19 | 0.32 | 0.00 | 0.00 |
| All | 112 | 41.06 | 0.44 | 0.77 | 0.29 | 1.00 | 0.10 | 0.17 | 0.06 | 0.23 |

The reference sequences of OPU107, OPU123 and OPU236 are *C. perfringens* ATCC13124 (CP000246), *P. russellii* (AY167952) and *E. coli* (X80725) respectively.

*: mean of inserted or deleted nucleotides in each sequence

SD: standard deviation
