# Supplementary material for: Metataxonomics reveal vultures as a reservoir for Clostridium perfringens
Source: Emerg Microbes Infect. 2017 Feb 22;6(2):e9–. doi: 10.1038/emi.2016.137 (PMC5322324; doi:10.1038/emi.2016.137)
Supplement: Supplementary Table 3 [file emi2016137x7.docx]

**Supplementary Table S3 List of OPUs affiliating with known species revealed***

| OPU name | OTUs | Taxon | Pacbio / Illunima | | | | | | | | | | Accession number (NCBI) |
| --- | --- | --- | --- | --- | --- | --- | --- | --- | --- | --- | --- | --- | --- |
|  |  |  | Am1 | Am2 | Am3 | Gb1 | Gb2 | Gb3 | Gh1 | Gh2 | Gh3 | Total |  |
| OPU001 | 171 / 0 | *Enterococcus clade 7* | 0.102 / 0 | 6.714 / 0 | 29.504 / 0 | 1.267 / 0 | 7.311 / 0 | 0 / 0 | 0 / 0 | 0 / 0 | 0.187 / 0 | **4.79 / 0** | AJ276354/AJ301830/Y17302/GU983697/AF061013/AF539705/EF197994/AJ271329 |
| OPU002 | 5 / 1 | *Enterococcus clade 4/5* | 0 / 0.002 | 0.014 / 0 | 0 / 0 | 0 / 0 | 0.225 / 0 | 0 / 0 | 0 / 0 | 0 / 0 | 0 / 0 | **0.028 / 0** | AJ888906/AF039903 /AF061007/AF039900 |
| OPU003 | 1 / 0 | *Enterococcus rivorum* | 0 / 0 | 0 / 0 | 0.019 / 0 | 0 / 0 | 0 / 0 | 0 / 0 | 0 / 0 | 0 / 0 | 0 / 0 | **0.002 / 0** | FN822765 |
| OPU004 | 10 / 1 | *Enterococcus faecalis* | 0 / 0.161 | 0.014 / 0 | 0 / 0 | 0.018 / 0 | 0.934 / 0 | 0 / 0 | 0 / 0 | 0 / 0 | 0.013 / 0 | **0.113 / 0.021** | AB012212 |
| OPU005 | 1 / 0 | *Catellicoccus marimammalium* | 0 / 0 | 0 / 0 | 0.019 / 0 | 0 / 0 | 0 / 0 | 0 / 0 | 0 / 0 | 0 / 0 | 0 / 0 | **0.002 / 0** | AJ854484 |
| OPU006 | 1 / 0 | *Granulicatella elegans* | 0 / 0 | 0.101 / 0 | 0 / 0 | 0 / 0 | 0 / 0 | 0 / 0 | 0 / 0 | 0 / 0 | 0 / 0 | **0.013 / 0** | AF016390 |
| OPU007 | 21 / 1 | *Streptococcus bovis group* | 0.179 / 0.214 | 0.752 / 0 | 0 / 0 | 0.018 / 0 | 2.126 / 0 | 0.071 / 0 | 0.032 / 0 | 0.015 / 0 | 0.187 / 0 | **0.393 / 0.028** | AJ301607/DQ232528/X94337/Z94012/AF177729/AF429763/AJ297215 |
| OPU008 | 1 / 0 | *Streptococcus thermophilus* | 0 / 0 | 0 / 0 | 0 / 0 | 0 / 0 | 0 / 0 | 0 / 0 | 0 / 0 | 0.029 / 0 | 0 / 0 | **0.004 / 0** | AY188354 |
| OPU009 | 6 / 0 | *Streptococcus mitis group* | 0 / 0 | 0.55 / 0 | 0 / 0 | 0 / 0 | 0 / 0 | 0 / 0 | 0 / 0 | 0 / 0 | 0.04 / 0 | **0.076 / 0** | HG315101/AY485603/AF003929/AY485602/AF003930/AY612844/AF003928/JN004270 |
| OPU010 | 2 / 0 | *Streptococcus ruminatorum* | 0 / 0 | 0 / 0 | 0 / 0 | 0 / 0 | 0 / 0 | 0 / 0 | 0 / 0 | 0.059 / 0 | 0 / 0 | **0.007 / 0** | AJ605748 |
| OPU011 | 2 / 0 | *Lactococcus lactis subsp* | 0.026 / 0 | 0.014 / 0 | 0 / 0 | 0 / 0 | 0 / 0 | 0 / 0 | 0 / 0 | 0 / 0 | 0 / 0 | **0.004 / 0** | AB100802/AB100804/AB100803/EU770697 |
| OPU012 | 3 / 0 | *Lactococcus garvieae* | 0 / 0 | 0.087 / 0 | 0 / 0 | 0 / 0 | 0.016 / 0 | 0 / 0 | 0 / 0 | 0 / 0 | 0 / 0 | **0.013 / 0** | AB598994 |
| OPU014 | 1 / 0 | *Lactobacillus brevis* | 0 / 0 | 0 / 0 | 0.019 / 0 | 0 / 0 | 0 / 0 | 0 / 0 | 0 / 0 | 0 / 0 | 0 / 0 | **0.002 / 0** | M58810 |
| OPU015 | 5 / 0 | *Lactobacillus reuteri* | 0 / 0 | 0.391 / 0 | 0 / 0 | 0 / 0 | 0 / 0 | 0 / 0 | 0 / 0 | 0 / 0 | 0.013 / 0 | **0.052 / 0** | L23507 |
| OPU017 | 23 / 7 | *Lactobacillus aviarius* | 0.204 / 0.23 | 0.072 / 0.042 | 1.115 / 0.654 | 0 / 0 | 0.161 / 0 | 0.232 / 0.018 | 0.016 / 0.018 | 0.059 / 0.072 | 0.067 / 0.013 | **0.196 / 0.082** | M58808 |
| OPU018 | 2 / 0 | *Lactobacillus salivarium group* | 0 / 0 | 0 / 0 | 0 / 0 | 0 / 0 | 0.016 / 0 | 0 / 0 | 0 / 0 | 0.015 / 0 | 0 / 0 | **0.004 / 0** | AB326350/AJ621554 |
| OPU020 | 1 / 0 | *Lactobacillus delbrueckii group(OPU020)* | 0 / 0 | 0 / 0 | 0 / 0 | 0.018 / 0 | 0 / 0 | 0 / 0 | 0 / 0 | 0 / 0 | 0 / 0 | **0.002 / 0** | AF519171/FR681902/AJ002515/EU487512 |
| OPU021 | 3 / 0 | *Lactobacillus rodentium* | 0 / 0 | 0.391 / 0 | 0 / 0 | 0.018 / 0 | 0 / 0 | 0 / 0 | 0 / 0 | 0 / 0 | 0.08 / 0 | **0.063 / 0** | HQ851022 |
| OPU022 | 1 / 0 | *Lactobacillus delbrueckii group(OPU022)* | 0 / 0 | 0 / 0 | 0 / 0 | 0 / 0 | 0.016 / 0 | 0 / 0 | 0 / 0 | 0 / 0 | 0 / 0 | **0.002 / 0** | FR681899/AJ306299/AY253657/FR681901 |
| OPU023 | 13 / 0 | *Bacillus subtilis group* | 0.026 / 0 | 0.13 / 0 | 0.056 / 0 | 0.018 / 0 | 0.789 / 0 | 0 / 0 | 0 / 0 | 0 / 0 | 0 / 0 | **0.116 / 0** | AJ831844/AJ831842/JX183147/AY876289/AF234854/AJ831841 |
| OPU024 | 7 / 0 | *Sporosarcina luteola/S.saromensis* | 0 / 0 | 0.232 / 0 | 0 / 0 | 0 / 0 | 0 / 0 | 0.071 / 0 | 0 / 0 | 0 / 0 | 0 / 0 | **0.037 / 0** | AB473560/AB243859 |
| OPU025 | 1 / 0 | *Planomicrobium chinense* | 0 / 0 | 0 / 0 | 0 / 0 | 0 / 0 | 0.016 / 0 | 0 / 0 | 0 / 0 | 0 / 0 | 0 / 0 | **0.002 / 0** | AJ697862 |
| OPU027 | 1 / 0 | *Lysinibacillus boronitolerans* | 0 / 0 | 0 / 0 | 0 / 0 | 0 / 0 | 0 / 0 | 0.018 / 0 | 0 / 0 | 0 / 0 | 0 / 0 | **0.002 / 0** | AB199591 |
| OPU028 | 1 / 0 | *Staphylococcus haemolyticus* | 0 / 0 | 0 / 0 | 0 / 0 | 0 / 0 | 0 / 0 | 0 / 0 | 0 / 0 | 0 / 0 | 0.013 / 0 | **0.002 / 0** | X66100 |
| OPU029 | 1 / 0 | *Staphylococcus saccharolyticus* | 0 / 0 | 0 / 0 | 0 / 0 | 0 / 0 | 0.016 / 0 | 0 / 0 | 0 / 0 | 0 / 0 | 0 / 0 | **0.002 / 0** | L37602 |
| OPU030 | 1 / 0 | *Staphylococcus equorum subsp* | 0 / 0 | 0 / 0 | 0 / 0 | 0 / 0 | 0.016 / 0 | 0 / 0 | 0 / 0 | 0 / 0 | 0 / 0 | **0.002 / 0** | AB009939/AF527483 |
| OPU031 | 2 / 0 | *Tumebacillus ginsengisoli* | 0 / 0 | 0 / 0 | 0 / 0 | 0 / 0 | 0.032 / 0 | 0 / 0 | 0 / 0 | 0 / 0 | 0 / 0 | **0.004 / 0** | AB245375 |
| OPU032 | 1 / 0 | *Gemella haemolysans* | 0 / 0 | 0 / 0 | 0.019 / 0 | 0 / 0 | 0 / 0 | 0 / 0 | 0 / 0 | 0 / 0 | 0 / 0 | **0.002 / 0** | L14326 |
| OPU033 | 1 / 0 | *Turicibacter sanguinis* | 0 / 0 | 0.014 / 0 | 0 / 0 | 0 / 0 | 0 / 0 | 0 / 0 | 0 / 0 | 0 / 0 | 0 / 0 | **0.002 / 0** | KM823691 |
| OPU039 | 1 / 0 | *Clostridium innocuum* | 0.026 / 0 | 0 / 0 | 0 / 0 | 0 / 0 | 0 / 0 | 0 / 0 | 0 / 0 | 0 / 0 | 0 / 0 | **0.002 / 0** | M23732 |
| OPU043 | 12 / 0 | *Clostridium ramosum* | 3.654 / 0 | 0.101 / 0 | 0 / 0 | 0 / 0 | 0 / 0 | 0 / 0 | 0 / 0 | 0 / 0 | 0.307 / 0 | **0.32 / 0** | X73440 |
| OPU056 | 1 / 0 | *Intestinimonas butyriciproducens* | 0 / 0 | 0.014 / 0 | 0 / 0 | 0 / 0 | 0 / 0 | 0 / 0 | 0 / 0 | 0 / 0 | 0 / 0 | **0.002 / 0** | KC311367 |
| OPU066 | 1 / 0 | *Ruminococcus albus* | 0 / 0 | 0 / 0 | 0 / 0 | 0 / 0 | 0.016 / 0 | 0 / 0 | 0 / 0 | 0 / 0 | 0 / 0 | **0.002 / 0** | L76598 |
| OPU100 | 26 / 8 | *Fusobacterium clade Ⅲ* | 4.446 / 9.529 | 0.014 / 0.022 | 0.019 / 0 | 0 / 0 | 0.048 / 0 | 0 / 0 | 0 / 0 | 0 / 0 | 0 / 0 | **0.331 / 1.237** | X55412/AJ867036/EU772816/HQ790099/HQ793785/HQ793813/HQ796283/HK241097 |
| OPU102 | 1 / 2 | *Cetobacterium somerae* | 0.051 / 0.422 | 0 / 0 | 0 / 0 | 0 / 0 | 0 / 0 | 0 / 0 | 0 / 0 | 0 / 0 | 0 / 0 | **0.004 / 0.055** | AJ438155 |
| OPU107 | 826 / 76 | *Clostridum perfringens* | 5.238 / 9.609 | 62.885 / 55.196 | 27.812 / 42.417 | 71.886 / 66.785 | 12.367 / 55.175 | 1.853 / 0.176 | 1.209 / 1.205 | 52.244 / 38.826 | 28.796 / 20.543 | **30.804 / 32.511** | CP000246 |
| OPU108 | 285 / 1 | *Eubacterium moniliforme/E.multiforme* | 2.044 / 0 | 0.969 / 0 | 10.857 / 0 | 16.618 / 0 | 5.298 / 0 | 6.895 / 0.003 | 4.962 / 0 | 0.221 / 0 | 8.652 / 0 | **6.172 / 0** | AB540985/AB018184 |
| OPU109 | 8 / 2 | *Clostridium baratii* | 0.026 / 0 | 0.014 / 0 | 0 / 0 | 0.036 / 0 | 0.193 / 0.725 | 0 / 0 | 0 / 0 | 0 / 0 | 0.093 / 0.039 | **0.042 / 0.097** | X68174 |
| OPU110 | 4 / 2 | *Clostridium disporicum/C.celatum* | 0.026 / 0.048 | 0 / 0 | 0 / 0 | 0.543 / 0.384 | 0 / 0 | 0 / 0 | 0 / 0 | 0 / 0 | 0 / 0 | **0.057 / 0.057** | Y18176 / X77844 |
| OPU111 | 1 / 2 | *Clostridium tertium* | 0 / 0.151 | 0 / 0 | 0 / 0 | 0 / 0 | 0 / 0 | 0 / 0 | 0 / 0 | 0 / 0 | 0.053 / 0.079 | **0.007 / 0.027** | Y18174 |
| OPU112 | 1 / 1 | *Clostridium septicum* | 0 / 0 | 0 / 0 | 0 / 0 | 0 / 0 | 0 / 0 | 0 / 0 | 0 / 0 | 0.015 / 0.055 | 0 / 0 | **0.002 / 0.005** | U59278 |
| OPU114 | 15 / 8 | *Clostridium paraputrificum* | 0.255 / 0.65 | 0.043 / 0 | 0 / 0 | 0.054 / 0.414 | 0.032 / 0.029 | 0.036 / 0.079 | 0 / 0.048 | 0 / 0 | 0.653 / 0.317 | **0.127 / 0.186** | X75907 |
| OPU115 | 2 / 0 | *Clostridium colicanis* | 0 / 0 | 0 / 0 | 0 / 0 | 0 / 0 | 0 / 0 | 0 / 0 | 0 / 0 | 0 / 0 | 0.04 / 0 | **0.006 / 0** | AJ420008 |
| OPU117 | 1 / 1 | *Clostridium putrefaciens* | 0 / 0 | 0 / 0 | 0 / 0 | 0 / 0 | 0.032 / 0.022 | 0 / 0 | 0 / 0 | 0 / 0 | 0 / 0 | **0.004 / 0.003** | AF127024 |
| OPU118 | 2 / 0 | *Clostridium fallax* | 0 / 0 | 0.014 / 0 | 0 / 0 | 0 / 0 | 0 / 0 | 0.018 / 0 | 0 / 0 | 0 / 0 | 0 / 0 | **0.004 / 0** | M59088 |
| OPU119 | 10 / 1 | *Clostridium novyi* | 0 / 0 | 0.029 / 0 | 0.037 / 0 | 0 / 0 | 0.209 / 0.043 | 0.428 / 0 | 0 / 0 | 0 / 0 | 0 / 0 | **0.076 / 0.006** | AB045606 |
| OPU120 | 6 / 2 | *Clostridium haemolyticum* | 0 / 0 | 0 / 0 | 0 / 0 | 0 / 0 | 0 / 0 | 0 / 0 | 0 / 0 | 0.088 / 0.09 | 0.227 / 0.483 | **0.042 / 0.052** | AB037910 |
| OPU123 | 478 / 28 | *Peptostreptococcus russellii* | 21.078 / 30.757 | 0.26 / 0.112 | 3.793 / 0.094 | 0.054 / 0 | 38.357 / 0.012 | 63.371 / 0.403 | 65.474 / 0.924 | 0.162 / 0.007 | 2.506 / 2.716 | **20.883 / 4.408** | AY167952 |
| OPU132 | 1 / 1 | *Clostridium lavalense* | 0.026 / 0.009 | 0 / 0 | 0 / 0 | 0 / 0 | 0 / 0 | 0 / 0 | 0 / 0 | 0 / 0 | 0 / 0 | **0.002 / 0.001** | EF564277 |
| OPU136 | 5 / 1 | *Blautia coccoides* | 0.588 / 1.064 | 0 / 0 | 0 / 0 | 0 / 0 | 0 / 0 | 0 / 0 | 0 / 0 | 0 / 0 | 0 / 0 | **0.042 / 0.138** | AB571656 |
| OPU209 | 1 / 0 | *Cellulomonas carbonis* | 0 / 0 | 0 / 0 | 0 / 0 | 0 / 0 | 0 / 0 | 0 / 0 | 0 / 0 | 0 / 0 | 0.013 / 0 | 0.002 / 0 | HQ702749 |
| OPU211 | 3 / 0 | *Actinomyces marimammalium* | 0.051 / 0 | 0 / 0 | 0 / 0 | 0 / 0 | 0 / 0 | 0 / 0 | 0 / 0 | 0.015 / 0 | 0 / 0 | **0.006 / 0** | AJ276405 |
| OPU212 | 1 / 0 | *Arthrobacter tecti* | 0 / 0 | 0.014 / 0 | 0 / 0 | 0 / 0 | 0 / 0 | 0 / 0 | 0 / 0 | 0 / 0 | 0 / 0 | **0.002 / 0** | AJ639829 |
| OPU213 | 2 / 0 | *Arthrobacter sanguinis* | 0 / 0 | 0 / 0 | 0 / 0 | 0 / 0 | 0.113 / 0 | 0.036 / 0 | 0 / 0 | 0 / 0 | 0 / 0 | **0.017 / 0** | EU086805 |
| OPU214 | 1 / 0 | *Microbacterium lacticum* | 0 / 0 | 0 / 0 | 0 / 0 | 0 / 0 | 0.016 / 0 | 0 / 0 | 0 / 0 | 0 / 0 | 0 / 0 | **0.002 / 0** | X7741 |
| OPU215 | 1 / 0 | *Microbacterium esteraromaticum* | 0 / 0 | 0 / 0 | 0 / 0 | 0 / 0 | 0.016 / 0 | 0 / 0 | 0 / 0 | 0 / 0 | 0 / 0 | **0.002 / 0** | Y17231 |
| OPU217 | 2 / 0 | *Propionibacterium acnes* | 0 / 0 | 0.014 / 0 | 0 / 0 | 0 / 0 | 0 / 0 | 0.018 / 0 | 0 / 0 | 0 / 0 | 0 / 0 | **0.004 / 0** | AB042288 |
| OPU220 | 2 / 1 | *Eggerthella lenta* | 0.051 / 0.051 | 0 / 0 | 0 / 0 | 0 / 0 | 0 / 0 | 0 / 0 | 0 / 0 | 0 / 0 | 0 / 0 | **0.004 / 0.007** | AF292375 |
| OPU221 | 1 / 0 | *Parvibacter caecicola* | 0 / 0 | 0 / 0 | 0 / 0 | 0 / 0 | 0.016 / 0 | 0 / 0 | 0 / 0 | 0 / 0 | 0 / 0 | **0.002 / 0** | GQ456228 |
| OPU236 | 236 / 8 | *Escherichia coli* | 41.415 / 16.972 | 3.444 / 0.038 | 0 / 0 | 0.308 / 2.778 | 0.209 / 0.282 | 0.125 / 0 | 0 / 0 | 0.044 / 0 | 19.517 / 16.967 | **6.212 / 4.135** | X87025 |
| OPU237 | 1 / 0 | *Enterobacter cancerogenus* | 0 / 0 | 0 / 0 | 0 / 0 | 0 / 0 | 0.016 / 0 | 0 / 0 | 0 / 0 | 0 / 0 | 0 / 0 | **0.002 / 0** | Z96078 |
| OPU238 | 1 / 0 | *Serratia marcescens subsp* | 0 / 0 | 0 / 0 | 0 / 0 | 0 / 0 | 0 / 0 | 0 / 0 | 0 / 0 | 0 / 0 | 0.013 / 0 | **0.002 / 0** | AB061685/AJ233431 |
| OPU240 | 2 / 0 | *Serratia quinivorans* | 0 / 0 | 0.029 / 0 | 0 / 0 | 0 / 0 | 0 / 0 | 0 / 0 | 0 / 0 | 0 / 0 | 0 / 0 | **0.004 / 0** | AJ233435 |
| OPU241 | 10 / 5 | *Plesiomonas shigelloides* | 0 / 0 | 0.014 / 0 | 0 / 0.101 | 0 / 0 | 0.113 / 0 | 0.089 / 0.389 | 0.286 / 0.233 | 0 / 0 | 0.04 / 0.429 | **0.063 / 0.119** | X60418 |
| OPU242 | 1 / 0 | *Vibrio litoralis/V.rumoiensis* | 0 / 0 | 0 / 0 | 0 / 0 | 0 / 0 | 0 / 0 | 0 / 0 | 0 / 0 | 0 / 0 | 0.013 / 0 | **0.002 / 0** | DQ097523 |
| OPU243 | 3 / 0 | *Acinetobacter johnsoni/A.bouvetii* | 0 / 0 | 0.029 / 0 | 0 / 0 | 0 / 0 | 0 / 0 | 0.036 / 0 | 0 / 0 | 0 / 0 | 0 / 0 | **0.007 / 0** | Z93440 / AF509827 |
| OPU244 | 1 / 0 | *Acinetobacter schindleri* | 0 / 0 | 0 / 0 | 0 / 0 | 0 / 0 | 0 / 0 | 0.018 / 0 | 0 / 0 | 0 / 0 | 0 / 0 | **0.002 / 0** | AJ278311 |
| OPU245 | 5 / 0 | *Acinetobacter lwoffii* | 0 / 0 | 0.637 / 0 | 0 / 0 | 0 / 0 | 0.097 / 0 | 0 / 0 | 0 / 0 | 0 / 0 | 0 / 0 | **0.092 / 0** | X81665 |
| OPU246 | 1 / 0 | *Acinetobacter harbinensis* | 0 / 0 | 0 / 0 | 0 / 0 | 0 / 0 | 0 / 0 | 0.018 / 0 | 0 / 0 | 0 / 0 | 0 / 0 | **0.002 / 0** | KC843488 |
| OPU251 | 5 / 0 | *Vulcaniibacterium thermophilum* | 0 / 0 | 0.029 / 0 | 0.019 / 0 | 0 / 0 | 0.032 / 0 | 0 / 0 | 0 / 0 | 0 / 0 | 0 / 0 | **0.009 / 0** | JQ746036 |
| OPU252 | 1 / 0 | *Lysobacter xinjiangensis* | 0 / 0 | 0 / 0 | 0 / 0 | 0 / 0 | 0.016 / 0 | 0 / 0 | 0 / 0 | 0 / 0 | 0 / 0 | **0.002 / 0** | EU833988 |
| OPU255 | 1 / 0 | *Curvibacter gracilis* | 0 / 0 | 0 / 0 | 0 / 0 | 0 / 0 | 0.016 / 0 | 0 / 0 | 0 / 0 | 0 / 0 | 0 / 0 | **0.002 / 0** | AB109889 |
| OPU256 | 1 / 0 | *Acidovorax wautersii* | 0 / 0 | 0 / 0 | 0.019 / 0 | 0 / 0 | 0 / 0 | 0 / 0 | 0 / 0 | 0 / 0 | 0 / 0 | **0.002 / 0** | JQ946365 |
| OPU257 | 3 / 0 | *Schlegelella aquatica* | 0 / 0 | 0 / 0 | 0 / 0 | 0 / 0 | 0.129 / 0 | 0.036 / 0 | 0 / 0 | 0 / 0 | 0 / 0 | **0.018 / 0** | DQ417336 |
| OPU258 | 1 / 0 | *Massilia timonae* | 0 / 0 | 0.014 / 0 | 0 / 0 | 0 / 0 | 0 / 0 | 0 / 0 | 0 / 0 | 0 / 0 | 0 / 0 | **0.002 / 0** | U54470 |
| OPU259 | 2 / 0 | *Herbaspirillum chlorophenolicum* | 0 / 0 | 0 / 0 | 0 / 0 | 0 / 0 | 0.016 / 0 | 0.018 / 0 | 0 / 0 | 0 / 0 | 0 / 0 | **0.004 / 0** | FJ267649 |
| OPU261 | 1 / 0 | *Ralstonia pickettii* | 0 / 0 | 0.014 / 0 | 0 / 0 | 0 / 0 | 0 / 0 | 0 / 0 | 0 / 0 | 0 / 0 | 0 / 0 | **0.002 / 0** | AY741342 |
| OPU262 | 4 / 0 | *Achromobacter xylosoxidans* | 0 / 0 | 0.014 / 0 | 0 / 0 | 0 / 0 | 0.032 / 0 | 0 / 0 | 0 / 0 | 0.015 / 0 | 0.013 / 0 | **0.009 / 0** | HF586506 |
| OPU263 | 1 / 0 | *Bordetella petrii* | 0 / 0 | 0 / 0 | 0 / 0 | 0 / 0 | 0 / 0 | 0 / 0 | 0 / 0 | 0 / 0 | 0.013 / 0 | **0.002 / 0** | AJ249861 |
| OPU264 | 1 / 0 | *Pigmentiphaga litoralis* | 0 / 0 | 0 / 0 | 0 / 0 | 0 / 0 | 0.016 / 0 | 0 / 0 | 0 / 0 | 0 / 0 | 0 / 0 | **0.002 / 0** | EU583723 |
| OPU266 | 2 / 0 | *Neisseria sicca* | 0 / 0 | 0.014 / 0 | 0.019 / 0 | 0 / 0 | 0 / 0 | 0 / 0 | 0 / 0 | 0 / 0 | 0 / 0 | **0.004 / 0** | ACKO020000 |
| OPU268 | 58 / 0 | *Sphingomonas melonis/S.aquatilis* | 0.026 / 0 | 0.926 / 0 | 1.171 / 0 | 0.507 / 0 | 1.208 / 0 | 0.606 / 0 | 0.064 / 0 | 3.37 / 0 | 0.173 / 0 | **0.944 / 0** | AF131295 |
| OPU269 | 1 / 0 | *Sphingomonas aestuarii* | 0 / 0 | 0.014 / 0 | 0 / 0 | 0 / 0 | 0 / 0 | 0 / 0 | 0 / 0 | 0 / 0 | 0 / 0 | **0.002 / 0** | EF660755 |
| OPU270 | 1 / 0 | *Sphingomonas hankookensis/S.panni* | 0 / 0 | 0 / 0 | 0 / 0 | 0 / 0 | 0.016 / 0 | 0 / 0 | 0 / 0 | 0 / 0 | 0 / 0 | **0.002 / 0** | FJ194436 / AJ575818 |
| OPU271 | 14 / 0 | *Sphingomonas echinoides* | 0 / 0 | 0.101 / 0 | 0.056 / 0 | 0.036 / 0 | 0.048 / 0 | 0.463 / 0 | 0 / 0 | 0.294 / 0 | 0.053 / 0 | **0.12 / 0** | AB021370 |
| OPU272 | 2 / 0 | *Sphingomonas paucimobilis* | 0 / 0 | 0 / 0 | 0.037 / 0 | 0 / 0 | 0 / 0 | 0 / 0 | 0 / 0 | 0 / 0 | 0 / 0 | **0.004 / 0** | U37337 |
| OPU277 | 4 / 0 | *Pannonibacter phragmitetus* | 0 / 0 | 0 / 0 | 0 / 0 | 0 / 0 | 0.081 / 0 | 0.036 / 0 | 0 / 0 | 0 / 0 | 0 / 0 | **0.013 / 0** | AJ400704 |
| OPU278 | 1 / 1 | *Bradyrhizobium neotropicale* | 0 / 0 | 0 / 0.123 | 0 / 0 | 0 / 0 | 0.016 / 0 | 0 / 0 | 0 / 0 | 0 / 0 | 0 / 0 | **0.002 / 0.017** | AY624134 |
| OPU283 | 1 / 0 | *Roseomonas aestuarii* | 0 / 0 | 0 / 0 | 0 / 0 | 0 / 0 | 0.016 / 0 | 0 / 0 | 0 / 0 | 0 / 0 | 0 / 0 | **0.002 / 0** | FM244739 |
| OPU284 | 3 / 0 | *Azospirillum brasilense* | 0 / 0 | 0.072 / 0 | 0 / 0 | 0 / 0 | 0 / 0 | 0 / 0 | 0 / 0 | 0 / 0 | 0 / 0 | **0.009 / 0** | AY324110 |
| OPU285 | 5 / 0 | *Brevundimonas vesicularis* | 0 / 0 | 0.029 / 0 | 0.019 / 0 | 0 / 0 | 0.016 / 0 | 0.018 / 0 | 0 / 0 | 0 / 0 | 0 / 0 | **0.009 / 0** | AJ227786 |
| OPU297 | 16 / 2 | *Bacteroides xylanisolvens* | 7.077 / 4.064 | 0.014 / 0 | 0 / 0 | 0 / 0 | 0 / 0 | 0 / 0 | 0 / 0 | 0 / 0 | 0 / 0 | **0.514 / 0.526** | AM230650 |
| OPU301 | 1 / 0 | *Alloprevotella rava* | 0.026 / 0 | 0 / 0 | 0 / 0 | 0 / 0 | 0 / 0 | 0 / 0 | 0 / 0 | 0 / 0 | 0 / 0 | **0.002 / 0** | JQ039189 |
| OPU305 | 1 / 0 | *Chishuiella changwenlii* | 0 / 0 | 0 / 0 | 0 / 0 | 0 / 0 | 0.016 / 0 | 0 / 0 | 0 / 0 | 0 / 0 | 0 / 0 | **0.002 / 0** | KF694751 |
| OPU306 | 1 / 0 | *Weeksella virosa* | 0 / 0 | 0 / 0 | 0 / 0 | 0 / 0 | 0 / 0 | 0.018 / 0 | 0 / 0 | 0 / 0 | 0 / 0 | **0.002 / 0** | CP002455 |
| OPU307 | 1 / 0 | *Elizabethkingia anophelis* | 0 / 0 | 0.014 / 0 | 0 / 0 | 0 / 0 | 0 / 0 | 0 / 0 | 0 / 0 | 0 / 0 | 0 / 0 | **0.002 / 0** | EF426425 |
| OPU310 | 1 / 0 | *Sphingobacterium kitahiroshimense* | 0 / 0 | 0 / 0 | 0 / 0 | 0 / 0 | 0 / 0 | 0.018 / 0 | 0 / 0 | 0 / 0 | 0 / 0 | **0.002 / 0** | AB361248 |
| OPU311 | 2 / 0 | *Rufibacter roseus* | 0 / 0 | 0.029 / 0 | 0 / 0 | 0 / 0 | 0 / 0 | 0 / 0 | 0 / 0 | 0 / 0 | 0 / 0 | **0.004 / 0** | KM264303 |
| OPU312 | 1 / 0 | *Rufibacter immobilis* | 0 / 0 | 0 / 0 | 0 / 0 | 0 / 0 | 0.032 / 0 | 0 / 0 | 0 / 0 | 0 / 0 | 0 / 0 | **0.004 / 0** | HG316123 |
| OPU315 | 1 / 0 | *Heliimonas saccharivorans* | 0 / 0 | 0 / 0 | 0 / 0 | 0 / 0 | 0 / 0 | 0.036 / 0 | 0 / 0 | 0 / 0 | 0 / 0 | **0.004 / 0** | JX458466 |
| OPU316 | 1 / 0 | *Helicobacter aurati* | 0 / 0 | 0.014 / 0 | 0 / 0 | 0 / 0 | 0 / 0 | 0 / 0 | 0 / 0 | 0 / 0 | 0 / 0 | **0.002 / 0** | AF297868 |
| OPU319 | 1 / 0 | *Opitutus terrae* | 0 / 0 | 0 / 0 | 0 / 0 | 0 / 0 | 0.016 / 0 | 0 / 0 | 0 / 0 | 0 / 0 | 0 / 0 | **0.002 / 0** | AJ229235 |
| OPU287 | 16 / 0 | *Chloroplast* | 0 / 0 | 0.391 / 0 | 0.019 / 0 | 0 / 0 | 0 / 0 | 0.036 / 0 | 0.048 / 0 | 0.015 / 0 | 0.08 / 0 | **0.074 / 0** | EU161508 |
| Total 102 (except OPU287) | 2391 / 163 |  | 86.64 / 73.93 | 79.31 / 55.53 | 74.61 / 43.27 | 91.40 / 70.36 | 70.55 / 56.29 | 74.58 / 1.07 | 72.04 / 2.43 | 56.65 / 39.05 | 61.78 / 41.59 | 72.99 / 43.72 |  |
| Total 103 (plus OPU287) | 2407 / 163 |  | 86.64 / 73.93 | 79.70 / 55.53 | 74.62 / 43.27 | 91.40 / 70.36 | 70.55 / 56.29 | 74.61 / 1.07 | 72.09 / 2.43 | 56.66 / 39.05 | 61.86 / 41.59 | 73.07 / 43.72 |  |

*The results are given as percentage of their sequence contribution to each single sample
