# Supplementary material for: Metataxonomics reveal vultures as a reservoir for Clostridium perfringens
Source: Emerg Microbes Infect. 2017 Feb 22;6(2):e9–. doi: 10.1038/emi.2016.137 (PMC5322324; doi:10.1038/emi.2016.137)
Supplement: Supplementary Table 4 [file emi2016137x8.docx]

**Supplementary Table S4 List of OPUs affiliating with new lineages within known genera revealed by PacBio and Illumina, which can be interpreted as yet to be described species***

| OPU name | OTUs | Taxon | Pacbio / Illunima | | | | | | | | | | Accession number (NCBI) |
| --- | --- | --- | --- | --- | --- | --- | --- | --- | --- | --- | --- | --- | --- |
|  |  |  | Am1 | Am2 | Am3 | Gb1 | Gb2 | Gb3 | Gh1 | Gh2 | Gh3 | Total |  |
| OPU049 | 7 / 2 | *Oscillibacter sp.1* | 0 / 0 | 0.058 / 0.013 | 0.019 / 0 | 0.018 / 0 | 0.016 / 0 | 0 / 0 | 0 / 0 | 0 / 0.003 | 0 / 0 | **0.013 / 0.002** | JF750939 |
| OPU050 | 4 / 1 | *Oscillibacter sp.2* | 0 / 0 | 0.014 / 0.018 | 0 / 0 | 0.018 / 0 | 0 / 0 | 0 / 0 | 0 / 0 | 0 / 0 | 0.027 / 0 | **0.007 / 0.002** | EF602928 |
| OPU051 | 3 / 0 | *Oscillibacter sp.3* | 0 / 0 | 0.043 / 0 | 0 / 0 | 0 / 0 | 0 / 0 | 0 / 0 | 0 / 0 | 0 / 0 | 0.013 / 0 | **0.007 / 0** | DQ015246 |
| OPU052 | 5 / 0 | *Oscillibacter sp.4* | 0 / 0 | 0.014 / 0 | 0 / 0 | 0 / 0 | 0 / 0 | 0 / 0 | 0 / 0 | 0 / 0 | 0.053 / 0 | **0.009 / 0** | EU510821 |
| OPU053 | 7 / 0 | *Oscillibacter sp.5* | 0 / 0 | 0.072 / 0 | 0 / 0 | 0 / 0 | 0.016 / 0 | 0 / 0 | 0 / 0 | 0 / 0 | 0.013 / 0 | **0.013 / 0** | FJ880098 |
| OPU054 | 1 / 0 | *Oscillibacter sp.6* | 0 / 0 | 0.014 / 0 | 0 / 0 | 0 / 0 | 0 / 0 | 0 / 0 | 0 / 0 | 0 / 0 | 0 / 0 | **0.002 / 0** | FJ880774 |
| OPU088 | 1 / 0 | *Butyricicoccus sp.* | 0 / 0 | 0.014 / 0 | 0 / 0 | 0 / 0 | 0 / 0 | 0 / 0 | 0 / 0 | 0 / 0 | 0 / 0 | **0.002 / 0** | L34618 |
| OPU090 | 9 / 1 | *Eubacterium sp.1* | 0 / 0 | 0.275 / 0.004 | 0.019 / 0 | 0.018 / 0 | 0 / 0 | 0 / 0 | 0 / 0 | 0.015 / 0 | 0.04 / 0 | **0.046 / 0** | L34625 |
| OPU098 | 155 / 2 | *Fusobacterium sp.1* | 0 / 0 | 0.145 / 0 | 1.803 / 0 | 0 / 0 | 22.979 / 0 | 20.363 / 0 | 26.622 / 0.378 | 0.015 / 0 | 0.427 / 0 | **8.098 / 0.045** | DQ113671 |
| OPU099 | 51 / 0 | *Fusobacterium sp.2* | 11.012 / 0 | 0.376 / 0 | 0 / 0 | 0 / 0 | 0.016 / 0 | 0 / 0 | 0.016 / 0 | 0 / 0 | 4.719 / 0 | **1.502 / 0** | HQ790981 |
| OPU101 | 1 / 0 | *Fusobacterium sp.3* | 0.026 / 0 | 0 / 0 | 0 / 0 | 0 / 0 | 0 / 0 | 0 / 0 | 0 / 0 | 0 / 0 | 0 / 0 | **0.002 / 0** | GQ448908 |
| OPU113 | 1 / 0 | *Clostridium sp.* | 0 / 0 | 0 / 0 | 0 / 0 | 0.018 / 0 | 0 / 0 | 0 / 0 | 0 / 0 | 0 / 0 | 0 / 0 | **0.002 / 0** | X68183/X71850 |
| OPU124_S | 156 / 65 | *Sporacetigenium sp.* | 0.153 / 1.034 | 2.879 / 40.676 | 14.668 / 38.922 | 2.788 / 5.493 | 1.916 / 8.965 | 1.746 / 0.15 | 0.064 / 0.226 | 2.487/ 25.157 | 2.333 / 4.496 | **3.164 / 12.352** | AY682207 |
| OPU148 | 1 / 0 | *Butyrivibrio sp.* | 0 / 0 | 0 / 0 | 0 / 0 | 0.018 / 0 | 0 / 0 | 0 / 0 | 0 / 0 | 0 / 0 | 0 / 0 | **0.002 / 0** | JQ084267 |
| OPU153 | 4 / 0 | *Roseburia sp.* | 0 / 0 | 0.014 / 0 | 0 / 0 | 0 / 0 | 0.016 / 0 | 0 / 0 | 0.016 / 0 | 0 / 0 | 0.013 / 0 | **0.007 / 0** | GU233441 |
| OPU175 | 18 / 6 | *Eubacterium sp.2* | 0.255 / 0.579 | 0.058 / 0 | 0.019 / 0.018 | 0 / 0 | 0.016 / 0 | 0.036 / 0.002 | 0.048 / 0.002 | 0.074 / 0 | 1.426 / 0.722 | **0.246 / 0.142** | HM596276 |
| OPU184 | 25 / 0 | *Peptoniphilus sp.* | 0.741 / 0 | 0.058 / 0 | 0.019 / 0 | 0.018 / 0 | 0.081 / 0 | 0.428 / 0 | 0.254 / 0 | 0.294 / 0 | 1.56 / 0 | **0.401 / 0** | GU440754 |
| OPU204 | 1 / 0 | *Pectinatus sp.* | 0 / 0 | 0 / 0 | 0 / 0 | 0 / 0 | 0.032 / 0 | 0 / 0 | 0 / 0 | 0 / 0 | 0 / 0 | 0.004 / 0 | HM108438 |
| OPU205 | 1 / 0 | *Selenomonas sp.* | 0 / 0 | 0 / 0 | 0 / 0 | 0 / 0 | 0.016 / 0 | 0 / 0 | 0 / 0 | 0 / 0 | 0 / 0 | 0.002 / 0 | LN613100 |
| OPU206 | 5 / 2 | *Veillonella sp.1* | 0 / 0 | 0 / 0.025 | 0.019 / 0 | 0 / 0 | 0 / 0 | 0 / 0 | 0 / 0 | 0.074 / 0.118 | 0.027 / 0 | 0.015 / 0.014 | AF186072 |
| OPU207 | 1 / 0 | *Veillonella sp.2* | 0 / 0 | 0 / 0 | 0.056 / 0 | 0 / 0 | 0 / 0 | 0 / 0 | 0 / 0 | 0 / 0 | 0 / 0 | 0.006 / 0 | EU778973 |
| OPU208 | 11 / 1 | *Veillonella sp.3* | 0 / 0 | 0 / 0 | 0 / 0 | 0 / 0 | 0.048 / 0 | 0 / 0 | 0.016 / 0 | 0.971 / 0.62 | 0 / 0 | 0.129 / 0.055 | EU778973 |
| OPU219 | 1 / 1 | *Paraeggerthella sp.* | 0 / 0 | 0 / 0 | 0 / 0 | 0 / 0 | 0 / 0 | 0 / 0 | 0 / 0 | 0 / 0 | 0.027 / 0.05 | **0.004 / 0.004** | JQ608168 |
| OPU223 | 5 / 0 | *Olsenella sp.1* | 0 / 0 | 0 / 0 | 0 / 0 | 0 / 0 | 0.048 / 0 | 0.036 / 0 | 0 / 0 | 0 / 0 | 0 / 0 | **0.009 / 0** | FN178463 |
| OPU224 | 1 / 0 | *Olsenella sp.2* | 0 / 0 | 0 / 0 | 0 / 0 | 0 / 0 | 0.016 / 0 | 0 / 0 | 0 / 0 | 0 / 0 | 0 / 0 | **0.002 / 0** | AB185601 |
| OPU225 | 1 / 0 | *Olsenella sp.3* | 0 / 0 | 0 / 0 | 0 / 0 | 0 / 0 | 0 / 0 | 0.018 / 0 | 0 / 0 | 0 / 0 | 0 / 0 | **0.002 / 0** | KC333946 |
| OPU233 | 1 / 0 | *Desulfovibrio sp.* | 0 / 0 | 0.014 / 0 | 0 / 0 | 0 / 0 | 0 / 0 | 0 / 0 | 0 / 0 | 0 / 0 | 0 / 0 | **0.002 / 0** | JN680672 |
| OPU247 | 18 / 0 | *Acinetobacter sp.* | 0.434 / 0 | 0 / 0 | 4.834 / 0 | 0.434 / 0 | 0 / 0 | 1.051 / 0 | 0.318 / 0 | 4.386 / 0 | 0 / 0 | **1.252 / 0** | X81659 |
| OPU248 | 15 / 0 | *Pseudomonas sp.* | 0.153 / 0 | 0.058 / 0 | 1.915 / 0 | 0.199 / 0 | 0 / 0 | 0.445 / 0 | 0.159 / 0 | 1.957 / 0 | 0 / 0 | **0.539 / 0** | D84009 |
| OPU249 | 4 / 0 | *Cellvibrio sp.* | 0 / 0 | 0.043 / 0 | 0 / 0 | 0.018 / 0 | 0.016 / 0 | 0 / 0 | 0 / 0 | 0 / 0 | 0 / 0 | **0.009 / 0** | AJ289164 |
| OPU250 | 1 / 0 | *Stenotrophomonas sp.* | 0 / 0 | 0.014 / 0 | 0 / 0 | 0 / 0 | 0 / 0 | 0 / 0 | 0 / 0 | 0 / 0 | 0 / 0 | **0.002 / 0** | AB021404 |
| OPU253 | 2 / 0 | *Comamonas sp.1* | 0 / 0 | 0 / 0 | 0 / 0 | 0 / 0 | 0.016 / 0 | 0.018 / 0 | 0 / 0 | 0 / 0 | 0 / 0 | **0.004 / 0** | AJ430344 |
| OPU254 | 1 / 0 | *Comamonas sp.2* | 0 / 0 | 0.014 / 0 | 0 / 0 | 0 / 0 | 0 / 0 | 0 / 0 | 0 / 0 | 0 / 0 | 0 / 0 | **0.002 / 0** | M11224 |
| OPU260 | 2 / 0 | *Noviherbaspirillum sp.* | 0 / 0 | 0.014 / 0 | 0 / 0 | 0.018 / 0 | 0 / 0 | 0 / 0 | 0 / 0 | 0 / 0 | 0 / 0 | **0.004 / 0** | DQ490985 |
| OPU265 | 9 / 2 | *Sutterella sp.* | 0 / 0.03 | 0.087 / 0 | 0.037 / 0.203 | 0 / 0 | 0.37 / 0 | 0 / 0 | 0 / 0 | 0 / 0 | 0.013 / 0 | **0.059 / 0.015** | AJ566849 |
| OPU275 | 7 / 0 | *Ochrobactrum sp.* | 0 / 0 | 0 / 0 | 0 / 0 | 0 / 0 | 0.467 / 0 | 0.232 / 0 | 0 / 0 | 0 / 0 | 0 / 0 | **0.078 / 0** | CP000758 |
| OPU298 | 3 / 7 | *Bacteroides sp.* | 0 / 0 | 0.029 / 0.043 | 0 / 0 | 0.018 / 0.005 | 0 / 0 | 0 / 0 | 0 / 0 | 0 / 0.018 | 0 / 0.06 | **0.006 / 0.014** | L16487 |
| OPU299 | 8 / 0 | *Prevotella sp.1* | 0 / 0 | 0.058 / 0 | 0 / 0 | 0 / 0 | 0.081 / 0 | 0 / 0 | 0 / 0 | 0.015 / 0 | 0 / 0 | **0.018 / 0** | AJ011682 |
| OPU300 | 4 / 0 | *Prevotella sp.2* | 0 / 0 | 0 / 0 | 0.019 / 0 | 0 / 0 | 0.016 / 0 | 0.018 / 0 | 0 / 0 | 0.015 / 0 | 0 / 0 | **0.007 / 0** | AB588018 |
| OPU302 | 2 / 0 | *Odoribacter sp.1* | 0 / 0 | 0.043 / 0 | 0 / 0 | 0 / 0 | 0 / 0 | 0 / 0 | 0 / 0 | 0 / 0 | 0 / 0 | **0.006 / 0** | AB606328 |
| OPU303 | 1 / 0 | Odoribacter sp.2 | 0 / 0 | 0 / 0 | 0 / 0 | 0 / 0 | 0 / 0 | 0 / 0 | 0 / 0 | 0 / 0 | 0.013 / 0 | 0.002 / 0 | AB490805 |
| OPU304 | 1 / 0 | *Flavobacterium sp.* | 0 / 0 | 0 / 0 | 0 / 0 | 0 / 0 | 0.016 / 0 | 0 / 0 | 0 / 0 | 0 / 0 | 0 / 0 | **0.002 / 0** | JX066803 |
| OPU308 | 14 / 0 | *Rikenellaceae sp.* | 0 / 0 | 0.203 / 0 | 0 / 0 | 0 / 0 | 0 / 0 | 0 / 0 | 0 / 0 | 0 / 0 | 0.013 / 0 | **0.028 / 0** | AY571428 |
| OPU309 | 6 / 8 | *Alistipes sp.* | 0 / 0 | 0.043 / 0.084 | 0 / 0 | 0 / 0.005 | 0.032 / 0.033 | 0 / 0 | 0 / 0 | 0.015 / 0 | 0 / 0.052 | **0.011 / 0.021** | AY643083 |
| OPU313_1 | 1 / 0 | *Adhaeribacter sp.1* | 0 / 0 | 0 / 0 | 0.019 / 0 | 0 / 0 | 0 / 0 | 0 / 0 | 0 / 0 | 0 / 0 | 0 / 0 | **0.002 / 0** | GQ421846 |
| OPU313_2 | 2 / 0 | *Adhaeribacter sp.2* | 0 / 0 | 0.029 / 0 | 0 / 0 | 0 / 0 | 0 / 0 | 0 / 0 | 0 / 0 | 0 / 0 | 0 / 0 | **0.004 / 0** | GQ421846 |
| OPU314 | 1 / 0 | *Flavisolibacter sp.* | 0 / 0 | 0 / 0 | 0.019 / 0 | 0 / 0 | 0 / 0 | 0 / 0 | 0 / 0 | 0 / 0 | 0 / 0 | **0.002 / 0** | AB267476 |
| OPU317 | 9 / 3 | *Helicobacter sp.* | 0 / 0 | 0.203 / 0.719 | 1.06 / 1.498 | 0 / 0 | 0.193 / 0 | 0 / 0 | 0 / 0 | 0.015 / 0.082 | 0 / 0 | **0.155 / 0.185** | DQ415546 |
| OPU318 | 1 / 0 | *Akkermansia sp.* | 0 / 0 | 0.014 / 0 | 0 / 0 | 0 / 0 | 0 / 0 | 0 / 0 | 0 / 0 | 0 / 0 | 0 / 0 | **0.002 / 0** | AY271254 |
| OPU320 | 4 / 0 | *Treponema sp.* | 0 / 0 | 0.029 / 0 | 0 / 0 | 0 / 0 | 0.032 / 0 | 0 / 0 | 0 / 0 | 0 / 0 | 0 / 0 | **0.007 / 0** | AY518274 |
| Total | 593 / 101 |  | 12.775 / 1.643 | 4.934 / 41.582 | 24.521 / 40.641 | 3.584 / 5.504 | 26.457 / 8.997 | 24.39 / 0.152 | 27.513 / 0.606 | 10.331/ 25.998 | 10.719 / 5.38 | 15.897 / 12.851 |  |

*The results are given as percentage of their sequence contribution to each single sample.
