# Supplementary material for: Metataxonomics reveal vultures as a reservoir for Clostridium perfringens
Source: Emerg Microbes Infect. 2017 Feb 22;6(2):e9–. doi: 10.1038/emi.2016.137 (PMC5322324; doi:10.1038/emi.2016.137)
Supplement: Supplementary Table 5 [file emi2016137x9.docx]

**Supplementary Table S5 List of OPUs affiliating with unknown lineages of uncultured representatives revealed by PacBio and Illumina***

| OPU name | OTUs | Taxon | Pacbio / Illunima | | | | | | | | | | accession number (NCBI) |
| --- | --- | --- | --- | --- | --- | --- | --- | --- | --- | --- | --- | --- | --- |
|  |  |  | Am1 | Am2 | Am3 | Gb1 | Gb2 | Gb3 | Gh1 | Gh2 | Gh3 | Total |  |
| OPU013 | 1 / 0 | *uncultured Aerococcaceae sp.* | 0 / 0 | 0 / 0 | 0 / 0 | 0 / 0 | 0.016 / 0 | 0 / 0 | 0 / 0 | 0 / 0 | 0 / 0 | **0.002 / 0** | D50541 |
| OPU016 | 3 / 2 | *uncultured lactobacillus* | 0 / 0 | 0 / 0 | 0 / 0 | 0 / 0 | 0.016 / 0.055 | 0 / 0 | 0 / 0 | 0.088 / 0.052 | 0 / 0 | **0.013 / 0.012** | AB3702332 |
| OPU019 | 6 / 0 | *uncultured lactobacillus* | 0 / 0 | 0.376 / 0 | 0 / 0 | 0 / 0 | 0 / 0 | 0 / 0 | 0 / 0 | 0 / 0 | 0.04 / 0 | **0.054 / 0** | Y16329 |
| OPU026 | 3 / 0 | *uncultured planomicrobium* | 0 / 0 | 0.058 / 0 | 0 / 0 | 0 / 0 | 0 / 0 | 0 / 0 | 0 / 0 | 0 / 0 | 0.013 / 0 | **0.009 / 0** | AF144750 |
| OPU034 | 2 / 0 | *uncultured ureaplasma* | 0 / 0 | 0 / 0 | 0.019 / 0 | 0 / 0 | 0.016 / 0 | 0 / 0 | 0 / 0 | 0 / 0 | 0 / 0 | **0.004 / 0** | JN792288 |
| OPU035 | 6 / 0 | *uncultured erysipelotrichaceae* | 0 / 0 | 0 / 0 | 0 / 0 | 0 / 0 | 0.048 / 0 | 0.036 / 0 | 0.048 / 0 | 0 / 0 | 0 / 0 | **0.015 / 0** | AF220064 |
| OPU036 | 1 / 0 | *uncultured erysipelotrichaceae* | 0 / 0 | 0.014 / 0 | 0 / 0 | 0 / 0 | 0 / 0 | 0 / 0 | 0 / 0 | 0 / 0 | 0 / 0 | **0.002 / 0** | HM124337 |
| OPU037 | 2 / 0 | *uncultured firmicutes* | 0 / 0 | 0 / 0 | 0 / 0 | 0 / 0 | 0.032 / 0 | 0 / 0 | 0 / 0 | 0 / 0 | 0 / 0 | **0.004 / 0** | FJ833617 |
| OPU038 | 1 / 0 | *uncultured erysipelotrichaceae* | 0 / 0 | 0.029 / 0 | 0 / 0 | 0 / 0 | 0 / 0 | 0 / 0 | 0 / 0 | 0 / 0 | 0 / 0 | **0.004 / 0** | AJ417075 |
| OPU040 | 5 / 2 | *uncultured anaeroplasmataceae* | 0 / 0 | 0.029 / 0 | 0 / 0 | 0.018 / 0.003 | 0 / 0 | 0 / 0 | 0 / 0 | 0.015 / 0.003 | 0.013 / 0 | **0.009 / 0.001** | JQ084129 |
| OPU041 | 2 / 1 | *uncultured firmicutes* | 0 / 0 | 0 / 0 | 0 / 0 | 0.018 / 0 | 0 / 0 | 0.018 / 0 | 0 / 0 | 0 / 0.003 | 0 / 0 | **0.004 / 0** | EU471804 |
| OPU042 | 94 / 11 | *uncultured firmicutes* | 0 / 0 | 0.145 / 0 | 0 / 0 | 4.562 / 6.408 | 0.612 / 14.126 | 0 / 0 | 0 / 0 | 29.831 / 30.626 | 0.067 / 0 | **4.308 / 5.389** | FM165584 |
| OPU044 | 1 / 0 | *uncultured firmicutes* | 0.026 / 0 | 0 / 0 | 0 / 0 | 0 / 0 | 0 / 0 | 0 / 0 | 0 / 0 | 0 / 0 | 0 / 0 | **0.002 / 0** | AJ621549/AB210824 |
| OPU045 | 19 / 0 | *uncultured firmicutes* | 0 / 0 | 0.159 / 0 | 0 / 0 | 0 / 0 | 0.048 / 0 | 0.018 / 0 | 0 / 0 | 0.029 / 0 | 0.04 / 0 | **0.037 / 0** | AB626909 |
| OPU046 | 14 / 1 | *uncultured firmicutes* | 0 / 0 | 0.492 / 0 | 0.019 / 0 | 0 / 0 | 0 / 0 | 0 / 0 | 0 / 0 | 0 / 0.003 | 0.013 / 0 | **0.067 / 0** | HW066368 |
| OPU047 | 8 / 0 | *uncultured firmicutes* | 0 / 0 | 0.072 / 0 | 0 / 0 | 0 / 0 | 0.016 / 0 | 0 / 0 | 0 / 0 | 0 / 0 | 0.027 / 0 | **0.015 / 0** | EU509962 |
| OPU048 | 1 / 1 | *uncultured firmicutes* | 0 / 0 | 0.014 / 0.012 | 0 / 0 | 0 / 0 | 0 / 0 | 0 / 0 | 0 / 0 | 0 / 0 | 0 / 0 | **0.002 / 0.002** | GU304152 |
| OPU055 | 57 / 1 | *uncultured clostridiales* | 0 / 0 | 1.244 / 0.007 | 0 / 0 | 0 / 0 | 0.064 / 0 | 0 / 0 | 0 / 0 | 0.044 / 0 | 0.067 / 0 | **0.181 / 0.001** | HW313929 |
| OPU057 | 1 / 0 | *uncultured clostridiales* | 0 / 0 | 0.014 / 0 | 0 / 0 | 0 / 0 | 0 / 0 | 0 / 0 | 0 / 0 | 0 / 0 | 0 / 0 | **0.002 / 0** | EF097979 |
| OPU058 | 1 / 0 | *uncultured clostridiales* | 0 / 0 | 0.014 / 0 | 0 / 0 | 0 / 0 | 0 / 0 | 0 / 0 | 0 / 0 | 0 / 0 | 0 / 0 | **0.002 / 0** | EU508780 |
| OPU059 | 3 / 0 | *uncultured clostridiales* | 0 / 0 | 0.058 / 0 | 0 / 0 | 0 / 0 | 0 / 0 | 0 / 0 | 0 / 0 | 0 / 0 | 0 / 0 | **0.007 / 0** | EU451602 |
| OPU060 | 3 / 2 | *uncultured clostridiales* | 0 / 0 | 0 / 0.013 | 0 / 0 | 0.018 / 0 | 0.016 / 0 | 0 / 0 | 0 / 0 | 0.029 / 0 | 0 / 0 | **0.007 / 0.002** | AB824398 |
| OPU061 | 7 / 0 | *uncultured ruminococcaceae* | 0 / 0 | 0.029 / 0 | 0 / 0 | 0 / 0 | 0.032 / 0 | 0 / 0 | 0 / 0 | 0.015 / 0 | 0.027 / 0 | **0.013 / 0** | AY991483 |
| OPU062 | 4 / 0 | *uncultured ruminococcaceae* | 0 / 0 | 0.043 / 0 | 0 / 0 | 0 / 0 | 0 / 0 | 0 / 0 | 0 / 0 | 0 / 0 | 0.013 / 0 | **0.007 / 0** | EU457369 |
| OPU063 | 4 / 1 | *uncultured ruminococcaceae* | 0 / 0 | 0.014 / 0.009 | 0 / 0 | 0 / 0 | 0.032 / 0 | 0 / 0 | 0 / 0 | 0.015 / 0 | 0 / 0 | **0.007 / 0.001** | EU472230 |
| OPU064 | 1 / 0 | *uncultured ruminococcaceae* | 0 / 0 | 0 / 0 | 0 / 0 | 0.018 / 0 | 0 / 0 | 0 / 0 | 0 / 0 | 0 / 0 | 0 / 0 | **0.002 / 0** | EU468594 |
| OPU065 | 1 / 0 | *uncultured ruminococcaceae* | 0 / 0 | 0.014 / 0 | 0 / 0 | 0 / 0 | 0 / 0 | 0 / 0 | 0 / 0 | 0 / 0 | 0 / 0 | **0.002 / 0** | EU462378 |
| OPU067 | 1 / 0 | *uncultured ruminococcaceae* | 0 / 0 | 0.014 / 0 | 0 / 0 | 0 / 0 | 0 / 0 | 0 / 0 | 0 / 0 | 0 / 0 | 0 / 0 | **0.002 / 0** | HM838988 |
| OPU068 | 3 / 1 | *uncultured clostridiaceae* | 0 / 0 | 0.029 / 0 | 0 / 0 | 0 / 0 | 0 / 0 | 0 / 0 | 0 / 0 | 0 / 0.003 | 0.013 / 0 | **0.006 / 0** | DQ777904 |
| OPU069 | 1 / 0 | *uncultured clostridiaceae* | 0 / 0 | 0 / 0 | 0 / 0 | 0 / 0 | 0.016 / 0 | 0 / 0 | 0 / 0 | 0 / 0 | 0 / 0 | **0.002 / 0** | EU843933 |
| OPU070 | 1 / 0 | *uncultured clostridiaceae* | 0 / 0 | 0.014 / 0 | 0 / 0 | 0 / 0 | 0 / 0 | 0 / 0 | 0 / 0 | 0 / 0 | 0 / 0 | **0.002 / 0** | HQ769937 |
| OPU071 | 1 / 0 | *uncultured clostridiaceae* | 0 / 0 | 0 / 0 | 0 / 0 | 0 / 0 | 0 / 0 | 0.018 / 0 | 0 / 0 | 0 / 0 | 0 / 0 | **0.002 / 0** | JX218663 |
| OPU072 | 1 / 0 | *uncultured clostridiaceae* | 0 / 0 | 0 / 0 | 0 / 0 | 0 / 0 | 0 / 0 | 0 / 0 | 0 / 0 | 0 / 0 | 0.013 / 0 | **0.002 / 0** | JQ993514 |
| OPU073 | 4 / 0 | *uncultured clostridiaceae* | 0 / 0 | 0.058 / 0 | 0 / 0 | 0 / 0 | 0 / 0 | 0 / 0 | 0.016 / 0 | 0 / 0 | 0 / 0 | **0.009 / 0** | AB506361 |
| OPU074 | 2 / 0 | *uncultured ruminococcaceae* | 0 / 0 | 0.014 / 0 | 0 / 0 | 0 / 0 | 0.016 / 0 | 0 / 0 | 0 / 0 | 0 / 0 | 0 / 0 | **0.004 / 0** | KM244841 |
| OPU075 | 1 / 0 | *uncultured clostridiaceae* | 0 / 0 | 0.014 / 0 | 0 / 0 | 0 / 0 | 0 / 0 | 0 / 0 | 0 / 0 | 0 / 0 | 0 / 0 | **0.002 / 0** | EU622687 |
| OPU076 | 1 / 0 | *uncultured clostridiaceae* | 0 / 0 | 0 / 0 | 0 / 0 | 0 / 0 | 0 / 0 | 0.018 / 0 | 0 / 0 | 0 / 0 | 0 / 0 | **0.002 / 0** | JX218545 |
| OPU077 | 2 / 1 | *uncultured clostridiaceae* | 0 / 0 | 0.014 / 0.002 | 0 / 0 | 0 / 0 | 0.016 / 0 | 0 / 0 | 0 / 0 | 0 / 0 | 0 / 0 | **0.004 / 0** | FJ881137 |
| OPU078 | 1 / 0 | *uncultured clostridiaceae* | 0 / 0 | 0 / 0 | 0 / 0 | 0 / 0 | 0.016 / 0 | 0 / 0 | 0 / 0 | 0 / 0 | 0 / 0 | **0.002 / 0** | EU773153 |
| OPU079 | 1 / 0 | *uncultured clostridiaceae* | 0 / 0 | 0 / 0 | 0 / 0 | 0 / 0 | 0.016 / 0 | 0 / 0 | 0 / 0 | 0 / 0 | 0 / 0 | **0.002 / 0** | EU469277 |
| OPU080 | 1 / 0 | *uncultured clostridiaceae* | 0 / 0 | 0 / 0 | 0 / 0 | 0.018 / 0 | 0 / 0 | 0 / 0 | 0 / 0 | 0 / 0 | 0 / 0 | **0.002 / 0** | FJ833505 |
| OPU081 | 4 / 1 | *uncultured ruminococcaceae* | 0 / 0 | 0.014 / 0 | 0 / 0 | 0.018 / 0 | 0.016 / 0.002 | 0 / 0 | 0 / 0 | 0 / 0 | 0.013 / 0 | **0.007 / 0** | Z49863 |
| OPU082 | 3 / 0 | *uncultured clostridiaceae* | 0 / 0 | 0.014 / 0 | 0 / 0 | 0 / 0 | 0.032 / 0 | 0 / 0 | 0 / 0 | 0 / 0 | 0 / 0 | **0.006 / 0** | KC162972 |
| OPU083 | 2 / 0 | *uncultured clostridiaceae* | 0 / 0 | 0.014 / 0 | 0 / 0 | 0 / 0 | 0.016 / 0 | 0 / 0 | 0 / 0 | 0 / 0 | 0 / 0 | **0.004 / 0** | AF371804 |
| OPU084 | 3 / 6 | *uncultured clostridiaceae* | 0 / 0 | 0.043 / 0.012 | 0 / 0 | 0 / 0 | 0 / 0.006 | 0 / 0 | 0 / 0 | 0 / 0 | 0.013 / 0.003 | **0.007 / 0.003** | EF434356 |
| OPU085 | 2 / 0 | *uncultured ruminococcaceae* | 0 / 0 | 0.014 / 0 | 0 / 0 | 0 / 0 | 0 / 0 | 0 / 0 | 0 / 0 | 0 / 0 | 0.013 / 0 | **0.004 / 0** | EU472099 |
| OPU086 | 1 / 0 | *uncultured clostridiales* | 0 / 0 | 0.014 / 0 | 0 / 0 | 0 / 0 | 0 / 0 | 0 / 0 | 0 / 0 | 0 / 0 | 0 / 0 | **0.002 / 0** | EU471556 |
| OPU087 | 5 / 1 | *uncultured clostridiaceae* | 0 / 0 | 0.087 / 0 | 0 / 0 | 0 / 0 | 0 / 0.002 | 0 / 0 | 0 / 0 | 0 / 0 | 0.013 / 0 | **0.013 / 0** | GQ448733 |
| OPU089 | 21 / 8 | *uncultured eubacteriaceae* | 0 / 0 | 0.188 / 0.094 | 0 / 0 | 0.018 / 0.004 | 0.016 / 0.012 | 0.036 / 0 | 0 / 0 | 0 / 0.015 | 0.093 / 0.009 | **0.044 / 0.017** | EU774625 |
| OPU091 | 2 / 2 | *uncultured eubacteriaceae* | 0 / 0 | 0.014 / 0.004 | 0 / 0 | 0 / 0.002 | 0 / 0 | 0 / 0 | 0 / 0 | 0 / 0 | 0.013 / 0 | **0.004 / 0.001** | DQ796951 |
| OPU092 | 5 / 1 | *uncultured clostridiaceae* | 0.077 / 0.112 | 0.043 / 0 | 0 / 0 | 0 / 0 | 0 / 0 | 0 / 0 | 0 / 0 | 0 / 0 | 0.013 / 0 | **0.013 / 0.015** | HW066373 |
| OPU093 | 7 / 0 | *uncultured clostridiales* | 0 / 0 | 0.101 / 0 | 0 / 0 | 0 / 0 | 0 / 0 | 0 / 0 | 0 / 0 | 0 / 0 | 0 / 0 | **0.013 / 0** | DQ815807 |
| OPU094 | 1 / 0 | *uncultured clostridiales* | 0 / 0 | 0 / 0 | 0 / 0 | 0 / 0 | 0 / 0 | 0 / 0 | 0 / 0 | 0 / 0 | 0.013 / 0 | **0.002 / 0** | JX222824 |
| OPU095 | 1 / 1 | *uncultured clostridiales* | 0 / 0 | 0 / 0 | 0 / 0 | 0 / 0 | 0 / 0 | 0 / 0 | 0 / 0 | 0 / 0.003 | 0.013 / 0 | **0.002 / 0** | JX222824 |
| OPU096 | 1 / 0 | *uncultured clostridiales* | 0 / 0 | 0 / 0 | 0 / 0 | 0 / 0 | 0.016 / 0 | 0 / 0 | 0 / 0 | 0 / 0 | 0 / 0 | **0.002 / 0** | KJ159287 |
| OPU097 | 1 / 0 | *uncultured firmicutes* | 0 / 0 | 0.014 / 0 | 0 / 0 | 0 / 0 | 0 / 0 | 0 / 0 | 0 / 0 | 0 / 0 | 0 / 0 | **0.002 / 0** | JX222824 |
| OPU103 | 1 / 0 | *uncultured fusobacteriales* | 0 / 0 | 0 / 0 | 0 / 0 | 0 / 0 | 0.016 / 0 | 0 / 0 | 0 / 0 | 0 / 0 | 0 / 0 | **0.002 / 0** | FJ470422 |
| OPU104 | 1 / 0 | *uncultured fusobacteriales* | 0 / 0 | 0 / 0 | 0 / 0 | 0 / 0 | 0 / 0 | 0.018 / 0 | 0 / 0 | 0 / 0 | 0 / 0 | **0.002 / 0** | FJ470422 |
| OPU105 | 1 / 0 | *uncultured fusobacteriales* | 0 / 0 | 0 / 0 | 0 / 0 | 0 / 0 | 0.016 / 0 | 0 / 0 | 0 / 0 | 0 / 0 | 0 / 0 | **0.002 / 0** | FJ470422 |
| OPU106 | 1 / 0 | *uncultured fusobacteriales* | 0 / 0 | 0 / 0 | 0 / 0 | 0 / 0 | 0 / 0 | 0.018 / 0 | 0 / 0 | 0 / 0 | 0 / 0 | **0.002 / 0** | FJ470422 |
| OPU116 | 27 / 94 | *uncultured clostridiales* | 0.026 / 4.814 | 0.029 / 1.183 | 0.093 / 15.195 | 0.091 / 16.955 | 0.145 / 12.398 | 0.036 / 7.655 | 0.032 / 8.254 | 0 / 0.175 | 0.067 / 7.084 | **0.057 / 7.992** | AM183047 |
| OPU121 | 1 / 0 | *uncultured clostridiales* | 0 / 0 | 0 / 0 | 0 / 0 | 0 / 0 | 0.016 / 0 | 0 / 0 | 0 / 0 | 0 / 0 | 0 / 0 | **0.002 / 0** | AM183047 |
| OPU122 | 6 / 44 | *uncultured clostridiales* | 0.026 / 0 | 0 / 0.007 | 0.019 / 0 | 0 / 0.08 | 0.064 / 0.02 | 0 / 48.519 | 0 / 42.946 | 0 / 0.026 | 0 / 0 | **0.011 / 10.985** | AM183047 |
| OPU124_U | 75 / 17 | *uncultured peptostreptococcaceae* | 0.051 / 0.515 | 0.506 / 0.212 | 0.353 / 0.651 | 0 / 0 | 0.097 / 0 | 0.071 / 0.39 | 0 / 0.131 | 0.633 / 1.009 | 8.266 / 7.744 | **1.347 / 0.98** | AM183047 |
| OPU125 | 87 / 0 | *uncultured Eisenbergiella_sp.* | 0 / 0 | 2.17 / 0 | 0 / 0 | 0.018 / 0 | 0.113 / 0 | 0 / 0 | 0 / 0 | 0.088 / 0 | 0.267 / 0 | **0.34 / 0** | KF814111 |
| OPU126 | 2 / 0 | *uncultured firmicutes* | 0 / 0 | 0.029 / 0 | 0 / 0 | 0 / 0 | 0 / 0 | 0 / 0 | 0 / 0 | 0 / 0 | 0 / 0 | **0.004 / 0** | DQ815536 |
| OPU127 | 23 / 0 | *uncultured firmicutes* | 0 / 0 | 0.42 / 0 | 0 / 0 | 0 / 0 | 0.016 / 0 | 0.018 / 0 | 0 / 0 | 0.015 / 0 | 0.067 / 0 | **0.068 / 0** | AB627654 |
| OPU128 | 31 / 1 | *uncultured firmicutes* | 0 / 0.012 | 0.304 / 0 | 0.019 / 0 | 0 / 0 | 0.097 / 0 | 0.071 / 0 | 0.016 / 0 | 0.029 / 0 | 0.013 / 0 | **0.067 / 0.002** | AB606350 |
| OPU129 | 12 / 5 | *uncultured firmicutes* | 0 / 0 | 0.13 / 0.043 | 0.019 / 0 | 0.018 / 0.027 | 0 / 0.006 | 0.018 / 0 | 0 / 0 | 0.029 / 0 | 0.04 / 0.011 | **0.031 / 0.011** | AB506313 |
| OPU130 | 15 / 2 | *uncultured firmicutes* | 0 / 0 | 0.217 / 0.008 | 0.019 / 0 | 0 / 0 | 0.016 / 0.006 | 0.018 / 0 | 0 / 0 | 0.029 / 0 | 0.08 / 0 | **0.048 / 0.002** | EU464217 |
| OPU131 | 5 / 0 | *uncultured clostridiales* | 0 / 0 | 0.043 / 0 | 0 / 0 | 0 / 0 | 0.016 / 0 | 0 / 0 | 0 / 0 | 0 / 0 | 0.013 / 0 | **0.009 / 0** | AB622837 |
| OPU133 | 1 / 0 | *uncultured clostridiales* | 0 / 0 | 0 / 0 | 0 / 0 | 0 / 0 | 0.016 / 0 | 0 / 0 | 0 / 0 | 0 / 0 | 0 / 0 | **0.002 / 0** | EF099816 |
| OPU134 | 1 / 0 | *uncultured clostridiales* | 0 / 0 | 0 / 0 | 0 / 0 | 0 / 0 | 0 / 0 | 0.018 / 0 | 0 / 0 | 0 / 0 | 0 / 0 | **0.002 / 0** | EF445150 |
| OPU135 | 2 / 0 | *uncultured clostridiales* | 0 / 0 | 0 / 0 | 0 / 0 | 0 / 0 | 0 / 0 | 0.018 / 0 | 0.016 / 0 | 0 / 0 | 0 / 0 | **0.004 / 0** | EU842510 |
| OPU137 | 4 / 0 | *uncultured clostridiales* | 0 / 0 | 0.029 / 0 | 0 / 0 | 0 / 0 | 0 / 0 | 0 / 0 | 0 / 0 | 0 / 0 | 0.027 / 0 | **0.007 / 0** | EU771550 |
| OPU138 | 3 / 0 | *uncultured clostridiales* | 0 / 0 | 0.029 / 0 | 0.019 / 0 | 0 / 0 | 0 / 0 | 0 / 0 | 0 / 0 | 0 / 0 | 0 / 0 | **0.006 / 0** | AF371574 |
| OPU139 | 1 / 0 | *uncultured clostridiales* | 0 / 0 | 0.014 / 0 | 0 / 0 | 0 / 0 | 0 / 0 | 0 / 0 | 0 / 0 | 0 / 0 | 0 / 0 | **0.002 / 0** | EU464384 |
| OPU140 | 2 / 1 | *uncultured clostridiales* | 0.026 / 0.023 | 0.014 / 0 | 0 / 0 | 0 / 0 | 0 / 0 | 0 / 0 | 0 / 0 | 0 / 0 | 0 / 0 | **0.004 / 0.003** | EF031542 |
| OPU141 | 1 / 0 | *uncultured clostridiales* | 0 / 0 | 0.014 / 0 | 0 / 0 | 0 / 0 | 0 / 0 | 0 / 0 | 0 / 0 | 0 / 0 | 0 / 0 | **0.002 / 0** | EU459675 |
| OPU142 | 5 / 0 | *uncultured clostridiales* | 0 / 0 | 0.058 / 0 | 0 / 0 | 0 / 0 | 0.016 / 0 | 0 / 0 | 0 / 0 | 0 / 0 | 0.027 / 0 | **0.013 / 0** | AB606340 |
| OPU143 | 3 / 0 | *uncultured clostridiales* | 0 / 0 | 0.058 / 0 | 0 / 0 | 0 / 0 | 0 / 0 | 0 / 0 | 0 / 0 | 0 / 0 | 0 / 0 | **0.007 / 0** | EU504127 |
| OPU144 | 1 / 0 | *uncultured clostridiales* | 0 / 0 | 0.014 / 0 | 0 / 0 | 0 / 0 | 0 / 0 | 0 / 0 | 0 / 0 | 0 / 0 | 0 / 0 | **0.002 / 0** | EU503877 |
| OPU145 | 1 / 0 | *uncultured clostridiales* | 0 / 0 | 0.014 / 0 | 0 / 0 | 0 / 0 | 0 / 0 | 0 / 0 | 0 / 0 | 0 / 0 | 0 / 0 | **0.002 / 0** | EU454610 |
| OPU146 | 3 / 0 | *uncultured clostridiales* | 0 / 0 | 0.014 / 0 | 0.019 / 0 | 0 / 0 | 0 / 0 | 0 / 0 | 0 / 0 | 0 / 0 | 0.013 / 0 | **0.006 / 0** | AB627725 |
| OPU147 | 16 / 5 | *uncultured clostridiales* | 0.179 / 0.39 | 0.014 / 0 | 0 / 0 | 0 / 0 | 0.016 / 0 | 0.036 / 0.019 | 0 / 0 | 0 / 0.011 | 0.84 / 0.432 | **0.137 / 0.093** | AF371573 |
| OPU149 | 1 / 1 | *uncultured clostridiales* | 0 / 0 | 0.029 / 0 | 0 / 0 | 0 / 0 | 0 / 0.002 | 0 / 0 | 0 / 0 | 0 / 0 | 0 / 0 | **0.004 / 0** | EU761670 |
| OPU150 | 1 / 0 | *uncultured clostridiales* | 0 / 0 | 0 / 0 | 0 / 0 | 0 / 0 | 0 / 0 | 0 / 0 | 0 / 0 | 0 / 0 | 0.013 / 0 | **0.002 / 0** | EU461131 |
| OPU151 | 2 / 0 | *uncultured clostridiales* | 0 / 0 | 0 / 0 | 0 / 0 | 0.018 / 0 | 0 / 0 | 0.018 / 0 | 0 / 0 | 0 / 0 | 0 / 0 | **0.004 / 0** | EU137610 |
| OPU152 | 8 / 1 | *uncultured clostridiales* | 0 / 0 | 0.058 / 0.004 | 0.019 / 0 | 0 / 0 | 0.032 / 0 | 0 / 0 | 0 / 0 | 0.015 / 0 | 0 / 0 | **0.015 / 0** | DQ801366 |
| OPU154 | 2 / 1 | *uncultured clostridiales* | 0 / 0 | 0.043 / 0 | 0 / 0 | 0 / 0 | 0 / 0 | 0.036 / 0 | 0 / 0 | 0 / 0.007 | 0 / 0 | **0.009 / 0.001** | DQ803905 |
| OPU155 | 2 / 0 | *uncultured clostridiales* | 0 / 0 | 0 / 0 | 0 / 0 | 0 / 0 | 0.032 / 0 | 0 / 0 | 0 / 0 | 0 / 0 | 0 / 0 | **0.004 / 0** | AB746796 |
| OPU156 | 5 / 0 | *uncultured clostridiales sp.* | 0 / 0 | 0 / 0 | 0 / 0 | 0 / 0 | 0.048 / 0 | 0.018 / 0 | 0.048 / 0 | 0 / 0 | 0 / 0 | **0.013 / 0** | AB494822 |
| OPU157 | 8 / 0 | *uncultured clostridiales* | 0 / 0 | 0.174 / 0 | 0 / 0 | 0 / 0 | 0 / 0 | 0 / 0 | 0 / 0 | 0 / 0 | 0.04 / 0 | **0.028 / 0** | HM123976 |
| OPU158 | 4 / 0 | *uncultured clostridiales* | 0 / 0 | 0.058 / 0 | 0 / 0 | 0 / 0 | 0 / 0 | 0 / 0 | 0 / 0 | 0 / 0 | 0 / 0 | **0.007 / 0** | FJ880993 |
| OPU159 | 4 / 0 | *uncultured clostridiales* | 0 / 0 | 0.058 / 0 | 0 / 0 | 0.018 / 0 | 0 / 0 | 0 / 0 | 0 / 0 | 0 / 0 | 0 / 0 | **0.009 / 0** | EU622706 |
| OPU160 | 2 / 0 | *uncultured clostridiales* | 0 / 0 | 0.043 / 0 | 0 / 0 | 0 / 0 | 0 / 0 | 0 / 0 | 0 / 0 | 0 / 0 | 0 / 0 | **0.006 / 0** | FJ881154 |
| OPU161 | 1 / 0 | *uncultured clostridiales* | 0 / 0 | 0.029 / 0 | 0 / 0 | 0 / 0 | 0 / 0 | 0 / 0 | 0 / 0 | 0 / 0 | 0 / 0 | **0.004 / 0** | AB192033 |
| OPU162 | 1 / 0 | *uncultured clostridiales* | 0 / 0 | 0.029 / 0 | 0 / 0 | 0 / 0 | 0 / 0 | 0 / 0 | 0 / 0 | 0 / 0 | 0 / 0 | **0.004 / 0** | DQ815773 |
| OPU163 | 1 / 1 | *uncultured clostridiales* | 0.026 / 0 | 0 / 0 | 0 / 0 | 0 / 0 | 0 / 0 | 0 / 0 | 0 / 0 | 0 / 0 | 0 / 0.003 | **0.002 / 0** | EU774315 |
| OPU164 | 5 / 0 | *uncultured clostridiales* | 0 / 0 | 0.058 / 0 | 0 / 0 | 0 / 0 | 0 / 0 | 0 / 0 | 0 / 0 | 0 / 0 | 0.013 / 0 | **0.009 / 0** | EU510644 |
| OPU165 | 4 / 0 | *uncultured clostridiales* | 0 / 0 | 0.072 / 0 | 0 / 0 | 0 / 0 | 0 / 0 | 0 / 0 | 0 / 0 | 0 / 0 | 0.013 / 0 | **0.011 / 0** | DQ777950 |
| OPU166 | 1 / 0 | *uncultured clostridiales* | 0 / 0 | 0 / 0 | 0 / 0 | 0 / 0 | 0 / 0 | 0 / 0 | 0 / 0 | 0 / 0 | 0.013 / 0 | **0.002 / 0** | AB555377 |
| OPU167 | 1 / 1 | *uncultured clostridiales* | 0 / 0 | 0.014 / 0.004 | 0 / 0 | 0 / 0 | 0 / 0 | 0 / 0 | 0 / 0 | 0 / 0 | 0 / 0 | **0.002 / 0** | HQ779750 |
| OPU168 | 1 / 1 | *uncultured clostridiales* | 0 / 0 | 0.014 / 0.003 | 0 / 0 | 0 / 0 | 0 / 0 | 0 / 0 | 0 / 0 | 0 / 0 | 0 / 0 | **0.002 / 0** | AY442828 |
| OPU169 | 2 / 0 | *uncultured clostridiales* | 0 / 0 | 0 / 0 | 0 / 0 | 0 / 0 | 0 / 0 | 0 / 0 | 0 / 0 | 0 / 0 | 0.027 / 0 | **0.004 / 0** | JF175077 |
| OPU170 | 1 / 0 | *uncultured clostridiales* | 0 / 0 | 0 / 0 | 0 / 0 | 0 / 0 | 0 / 0 | 0.018 / 0 | 0 / 0 | 0 / 0 | 0 / 0 | **0.002 / 0** | JF175077 |
| OPU171 | 1 / 0 | *uncultured clostridiales* | 0 / 0 | 0 / 0 | 0 / 0 | 0 / 0 | 0 / 0 | 0 / 0 | 0.032 / 0 | 0 / 0 | 0 / 0 | **0.004 / 0** | EU844116 |
| OPU172 | 9 / 0 | *uncultured clostridiales sp.* | 0 / 0 | 0.116 / 0 | 0 / 0 | 0 / 0 | 0 / 0 | 0 / 0 | 0 / 0 | 0 / 0 | 0.027 / 0 | **0.018 / 0** | EF096137 |
| OPU173 | 10 / 2 | *uncultured Clostridiales sp.* | 0 / 0 | 0.043 / 0.01 | 0.019 / 0 | 0 / 0 | 0.016 / 0.006 | 0.036 / 0 | 0.032 / 0 | 0 / 0 | 0.013 / 0 | **0.018 / 0.002** | EF099559 |
| OPU174 | 1 / 0 | *uncultured clostridiales* | 0 / 0 | 0 / 0 | 0 / 0 | 0 / 0 | 0 / 0 | 0 / 0 | 0 / 0 | 0.015 / 0 | 0 / 0 | **0.002 / 0** | JQ248091 |
| OPU176 | 1 / 3 | *uncultured clostridiales* | 0 / 0.045 | 0 / 0.004 | 0 / 0 | 0 / 0 | 0.016 / 0 | 0 / 0 | 0 / 0 | 0 / 0 | 0 / 0 | **0.002 / 0.006** | EU842597 |
| OPU177 | 1 / 1 | *uncultured clostridiales* | 0 / 0 | 0 / 0 | 0 / 0 | 0 / 0 | 0 / 0.002 | 0.018 / 0 | 0 / 0 | 0 / 0 | 0 / 0 | **0.002 / 0** | EU843849 |
| OPU178 | 1 / 0 | *uncultured clostridiales* | 0 / 0 | 0.014 / 0 | 0 / 0 | 0 / 0 | 0 / 0 | 0 / 0 | 0 / 0 | 0 / 0 | 0 / 0 | **0.002 / 0** | AF287762 |
| OPU179 | 1 / 0 | *uncultured clostridiales* | 0 / 0 | 0.014 / 0 | 0 / 0 | 0 / 0 | 0 / 0 | 0 / 0 | 0 / 0 | 0 / 0 | 0 / 0 | **0.002 / 0** | EU450964 |
| OPU180 | 18 / 3 | *uncultured clostridiales sp.* | 0 / 0 | 0.072 / 0.003 | 0 / 0 | 0 / 0 | 0.081 / 0 | 0.107 / 0.02 | 0.032 / 0 | 0 / 0 | 0.027 / 0 | **0.037 / 0.003** | AB506428 |
| OPU181 | 14 / 0 | *uncultured clostridiales* | 0 / 0 | 0.391 / 0 | 0 / 0 | 0 / 0 | 0 / 0 | 0 / 0 | 0 / 0 | 0 / 0 | 0.04 / 0 | **0.055 / 0** | AB671763 |
| OPU182 | 1 / 0 | *uncultured clostridiales* | 0 / 0 | 0.014 / 0 | 0 / 0 | 0 / 0 | 0 / 0 | 0 / 0 | 0 / 0 | 0 / 0 | 0 / 0 | **0.002 / 0** | EU466370 |
| OPU183 | 29 / 4 | *uncultured clostridiales sp.* | 0 / 0 | 0.232 / 0.003 | 0.019 / 0 | 0.018 / 0 | 0.032 / 0.007 | 0.053 / 0 | 0 / 0 | 0.029 / 0 | 0.107 / 0 | **0.061 / 0.001** | AB824307 |
| OPU185 | 2 / 2 | *uncultured clostridiales* | 0 / 0.007 | 0 / 0 | 0 / 0 | 0 / 0 | 0 / 0 | 0.018 / 0 | 0 / 0 | 0 / 0 | 0.04 / 0.012 | **0.007 / 0.002** | AB298735 |
| OPU186 | 1 / 0 | *uncultured clostridiales* | 0 / 0 | 0 / 0 | 0 / 0 | 0 / 0 | 0 / 0 | 0 / 0 | 0.016 / 0 | 0 / 0 | 0 / 0 | **0.002 / 0** | EU474559 |
| OPU187 | 1 / 0 | *uncultured firmicutes* | 0 / 0 | 0 / 0 | 0 / 0 | 0 / 0 | 0 / 0 | 0 / 0 | 0.016 / 0 | 0 / 0 | 0 / 0 | **0.002 / 0** | EU381710 |
| OPU188 | 4 / 0 | *uncultured firmicutes* | 0 / 0 | 0.014 / 0 | 0 / 0 | 0 / 0 | 0.032 / 0 | 0 / 0 | 0 / 0 | 0.015 / 0 | 0 / 0 | **0.007 / 0** | AF371827 |
| OPU189 | 3 / 0 | *uncultured firmicutes* | 0 / 0 | 0.072 / 0 | 0 / 0 | 0 / 0 | 0 / 0 | 0 / 0 | 0 / 0 | 0 / 0 | 0 / 0 | **0.009 / 0** | EU766256 |
| OPU190 | 10 / 9 | *uncultured firmicutes sp.* | 0 / 0 | 0 / 0.015 | 0 / 0 | 0.036 / 0.006 | 0 / 0.006 | 0.036 / 0 | 0.048 / 0 | 0 / 0.003 | 0.04 / 0 | **0.018 / 0.004** | AB606382 |
| OPU191 | 1 / 0 | *uncultured firmicutes* | 0 / 0 | 0.014 / 0 | 0 / 0 | 0 / 0 | 0 / 0 | 0 / 0 | 0 / 0 | 0 / 0 | 0 / 0 | **0.002 / 0** | EU451218 |
| OPU201 | 13 / 3 | *uncultured firmicutes* | 0.128 / 0.176 | 0.101 / 0 | 0 / 0 | 0 / 0 | 0 / 0 | 0 / 0 | 0 / 0 | 0 / 0 | 1.213 / 0.326 | **0.19 / 0.052** | AF481224 |
| OPU202 | 1 / 0 | *uncultured firmicutes* | 0 / 0 | 0.014 / 0 | 0 / 0 | 0 / 0 | 0 / 0 | 0 / 0 | 0 / 0 | 0 / 0 | 0 / 0 | 0.002 / 0 | GQ327472 |
| OPU203 | 139 / 8 | *uncultured veillonellaceae* | 0 / 0.071 | 1.418 / 0.179 | 0.112 / 0.214 | 0.036 / 0 | 0.129 / 0 | 0.053 / 0.034 | 0.048 / 0.021 | 2.001 / 2.229 | 14.771 / 7.889 | 2.52 / 0.96 | EU939429 |
| OPU210 | 1 / 0 | *uncultured actinobacteria* | 0 / 0 | 0 / 0 | 0 / 0 | 0 / 0 | 0 / 0 | 0 / 0 | 0 / 0 | 0 / 0 | 0.013 / 0 | **0.002 / 0** | EU778954 |
| OPU216 | 1 / 0 | *uncultured actinobacteria* | 0 / 0 | 0.014 / 0 | 0 / 0 | 0 / 0 | 0 / 0 | 0 / 0 | 0 / 0 | 0 / 0 | 0 / 0 | **0.002 / 0** | JX273674 |
| OPU218 | 1 / 3 | *uncultured bifidobacteriaceae* | 0.026 / 0.025 | 0 / 0.047 | 0 / 0.032 | 0 / 0 | 0 / 0 | 0 / 0 | 0 / 0 | 0 / 0 | 0 / 0 | **0.002 / 0.011** | JX863369 |
| OPU222 | 1 / 0 | *uncultured coriobacteriia* | 0 / 0 | 0.014 / 0 | 0 / 0 | 0 / 0 | 0 / 0 | 0 / 0 | 0 / 0 | 0 / 0 | 0 / 0 | **0.002 / 0** | DQ800299 |
| OPU226 | 5 / 1 | *uncultured coriobacteriales* | 0 / 0 | 0.014 / 0 | 0.056 / 0 | 0 / 0 | 0 / 0 | 0.018 / 0 | 0 / 0 | 0 / 0 | 0.053 / 0.125 | **0.017 / 0.011** | KJ734841 |
| OPU227 | 2 / 2 | *uncultured coriobacteriales* | 0 / 0.022 | 0 / 0 | 0 / 0 | 0 / 0 | 0 / 0 | 0 / 0 | 0 / 0 | 0 / 0 | 0.04 / 0.027 | **0.006 / 0.005** | JN680683 |
| OPU228 | 1 / 0 | *uncultured gemmatimonadales* | 0 / 0 | 0 / 0 | 0.019 / 0 | 0 / 0 | 0 / 0 | 0 / 0 | 0 / 0 | 0 / 0 | 0 / 0 | **0.002 / 0** | EU193062 |
| OPU229 | 87 / 0 | *uncultured firmicutes sp.* | 0 / 0 | 2.619 / 0 | 0 / 0 | 0 / 0 | 0.032 / 0 | 0 / 0 | 0 / 0 | 0 / 0 | 0.387 / 0 | **0.392 / 0** | GQ503874 |
| OPU230 | 1 / 0 | *uncultured deltaproteobacteria* | 0 / 0 | 0 / 0 | 0 / 0 | 0 / 0 | 0.016 / 0 | 0 / 0 | 0 / 0 | 0 / 0 | 0 / 0 | **0.002 / 0** | EF098374 |
| OPU231 | 1 / 0 | *uncultured deltaproteobacteria* | 0 / 0 | 0 / 0 | 0 / 0 | 0 / 0 | 0 / 0 | 0 / 0 | 0 / 0 | 0.015 / 0 | 0 / 0 | **0.002 / 0** | AY976442 |
| OPU232 | 1 / 0 | *uncultured deltaproteobacteria* | 0 / 0 | 0 / 0 | 0 / 0 | 0 / 0 | 0 / 0 | 0.018 / 0 | 0 / 0 | 0 / 0 | 0 / 0 | **0.002 / 0** | EU461101 |
| OPU234 | 3 / 0 | *uncultured deltaproteobacteria* | 0 / 0 | 0.043 / 0 | 0 / 0 | 0 / 0 | 0 / 0 | 0 / 0 | 0 / 0 | 0 / 0 | 0 / 0 | **0.006 / 0** | EU453154 |
| OPU235 | 1 / 0 | *uncultured acidobacteria* | 0 / 0 | 0 / 0 | 0 / 0 | 0 / 0 | 0.032 / 0 | 0 / 0 | 0 / 0 | 0 / 0 | 0 / 0 | **0.004 / 0** | HQ003628 |
| OPU239 | 1 / 0 | *uncultured enterobacteriaceae* | 0 / 0 | 0 / 0 | 0 / 0 | 0 / 0 | 0 / 0 | 0 / 0 | 0 / 0 | 0 / 0 | 0.013 / 0 | **0.002 / 0** | KF057941/KF057944 |
| OPU267 | 1 / 0 | *uncultured cystobacteraceae* | 0 / 0 | 0.014 / 0 | 0 / 0 | 0 / 0 | 0 / 0 | 0 / 0 | 0 / 0 | 0 / 0 | 0 / 0 | **0.002 / 0** | LN563572 |
| OPU273 | 1 / 0 | *uncultured sphingomonadales* | 0 / 0 | 0.014 / 0 | 0 / 0 | 0 / 0 | 0 / 0 | 0 / 0 | 0 / 0 | 0 / 0 | 0 / 0 | **0.002 / 0** | DQ011529/AF465836 |
| OPU274 | 1 / 0 | *uncultured alphaproteobacteria* | 0 / 0 | 0.014 / 0 | 0 / 0 | 0 / 0 | 0 / 0 | 0 / 0 | 0 / 0 | 0 / 0 | 0 / 0 | **0.002 / 0** | HF565048 |
| OPU276 | 1 / 1 | *uncultured rhizobiaceae* | 0 / 0 | 0 / 0.004 | 0 / 0 | 0 / 0 | 0.016 / 0 | 0 / 0 | 0 / 0 | 0 / 0 | 0 / 0 | **0.002 / 0** | EF516927 |
| OPU279 | 7 / 0 | *uncultured paracoccus* | 0 / 0 | 0.318 / 0 | 0 / 0 | 0.036 / 0 | 0.064 / 0 | 0 / 0 | 0 / 0 | 0 / 0 | 0 / 0 | **0.052 / 0** | JF159680 |
| OPU280 | 1 / 0 | *uncultured paracoccus* | 0 / 0 | 0.014 / 0 | 0 / 0 | 0 / 0 | 0 / 0 | 0 / 0 | 0 / 0 | 0 / 0 | 0 / 0 | **0.002 / 0** | EU660389 |
| OPU281 | 1 / 0 | *uncultured rhodobacterales* | 0 / 0 | 0.014 / 0 | 0 / 0 | 0 / 0 | 0 / 0 | 0 / 0 | 0 / 0 | 0 / 0 | 0 / 0 | **0.002 / 0** | FM874293 |
| OPU282 | 1 / 0 | *uncultured alphaproteobacteria* | 0 / 0 | 0.029 / 0 | 0 / 0 | 0 / 0 | 0 / 0 | 0 / 0 | 0 / 0 | 0 / 0 | 0 / 0 | **0.004 / 0** | JF181681 |
| OPU286 | 2 / 0 | *uncultured deferribacteraceae* | 0 / 0 | 0.058 / 0 | 0 / 0 | 0 / 0 | 0 / 0 | 0 / 0 | 0 / 0 | 0 / 0 | 0 / 0 | **0.007 / 0** | AF157057 |
| OPU288 | 3 / 1 | *uncultured bdellovibrionaceae* | 0 / 0 | 0 / 0.004 | 0.019 / 0 | 0 / 0 | 0 / 0 | 0 / 0 | 0 / 0 | 0.015 / 0 | 0.013 / 0 | **0.006 / 0.001** | GQ451253 |
| OPU289 | 12 / 6 | *uncultured firmicutes sp.* | 0 / 0.052 | 0.058 / 0 | 0 / 0 | 0 / 0 | 0.161 / 0 | 0.053 / 0.011 | 0 / 0.012 | 0 / 0 | 0 / 0 | **0.031 / 0.01** | EU459481 |
| OPU290 | 48 / 7 | *uncultured firmicutes sp.* | 0 / 0 | 0.695 / 0.025 | 0 / 0 | 0.018 / 0.006 | 0.081 / 0.002 | 0 / 0 | 0 / 0 | 0.015 / 0 | 0.12 / 0.003 | **0.118 / 0.005** | AB606322 |
| OPU291 | 15 / 0 | *uncultured bacteroidetes* | 0 / 0 | 0.116 / 0 | 0 / 0 | 0 / 0 | 0.161 / 0 | 0 / 0 | 0 / 0 | 0 / 0 | 0.027 / 0 | **0.037 / 0** | AY239461 |
| OPU292 | 18 / 0 | *uncultured bacteroidetes* | 0 / 0 | 0.232 / 0 | 0 / 0 | 0 / 0 | 0.048 / 0 | 0 / 0 | 0 / 0 | 0 / 0 | 0.08 / 0 | **0.046 / 0** | DQ815337 |
| OPU293 | 2 / 0 | *uncultured bacteroidetes* | 0 / 0 | 0 / 0 | 0 / 0 | 0 / 0 | 0.032 / 0 | 0 / 0 | 0 / 0 | 0 / 0 | 0 / 0 | **0.004 / 0** | FJ684475 |
| OPU294 | 11 / 0 | *uncultured bacteroidetes* | 0 / 0 | 0.145 / 0 | 0 / 0 | 0 / 0 | 0.064 / 0 | 0 / 0 | 0 / 0 | 0 / 0 | 0 / 0 | **0.026 / 0** | AY239400 |
| OPU295 | 1 / 0 | *uncultured bacteroidetes* | 0 / 0 | 0.029 / 0 | 0 / 0 | 0 / 0 | 0 / 0 | 0 / 0 | 0 / 0 | 0 / 0 | 0 / 0 | **0.004 / 0** | EU622696 |
| OPU296 | 7 / 0 | *uncultured bacteroidetes* | 0 / 0 | 0.029 / 0 | 0 / 0 | 0.018 / 0 | 0.064 / 0 | 0.018 / 0 | 0 / 0 | 0 / 0 | 0.013 / 0 | **0.017 / 0** | AB746793 |
| OPU321 | 1 / 0 | *uncultured planctomycetales* | 0 / 0 | 0 / 0 | 0 / 0 | 0 / 0 | 0.016 / 0 | 0 / 0 | 0 / 0 | 0 / 0 | 0 / 0 | **0.002 / 0** | AF239699 |
| Total | 1223/275 |  | 0.588 / 6.263 | 15.367 / 1.908 | 0.855 / 16.093 | 5.014 / 23.49 | 2.995 / 26.655 | 0.998 / 56.648 | 0.398 / 51.365 | 33.01 / 34.166 | 27.423/23.666 | 11.037/26.588 |  |

*The results are given as percentage of their sequence contribution to each single sample
