# Supplementary material for: Metataxonomics reveal vultures as a reservoir for Clostridium perfringens
Source: Emerg Microbes Infect. 2017 Feb 22;6(2):e9–. doi: 10.1038/emi.2016.137 (PMC5322324; doi:10.1038/emi.2016.137)
Supplement: Supplementary Table 6 [file emi2016137x10.docx]

**Supplementary Table S6 List of OPUs only affiliating with Illumina**

| OPU name | OTUs number | Taxon | Pacbio / Illunima | | | | | | | | | | accession number (NCBI) |
| --- | --- | --- | --- | --- | --- | --- | --- | --- | --- | --- | --- | --- | --- |
|  |  |  | Am1 | Am2 | Am3 | Gb1 | Gb2 | Gb3 | Gh1 | Gh2 | Gh3 | Total |  |
| OPU400 | 3 | *Proteiniclasticum ruminis* | 0.022 | 0.000 | 0.000 | 0.000 | 0.013 | 0.000 | 0.000 | 0.000 | 0.035 | 0.008 | DQ852338 |
| OPU401 | 2 | *Streptococcus thoraltensis* | 0.014 | 0.000 | 0.000 | 0.000 | 0.000 | 0.000 | 0.000 | 0.003 | 0.000 | 0.002 | Y09007 |
| OPU402 | 1 | *Leuconostoc mesenteroides* | 0.000 | 0.004 | 0.000 | 0.000 | 0.000 | 0.000 | 0.000 | 0.000 | 0.000 | 0.001 | CP000414 |
| OPU403 | 1 | *uncultured staphylococcaceae* | 0.000 | 0.000 | 0.000 | 0.000 | 0.003 | 0.000 | 0.000 | 0.000 | 0.000 | 0.000 | EU240886/Y15712 |
| OPU404 | 2 | *uncultured firmicutes* | 0.636 | 0.000 | 0.000 | 0.000 | 0.000 | 0.000 | 0.000 | 0.000 | 0.034 | 0.085 | DQ799536 |
| OPU405 | 1 | *uncultured erysipelotrichaceae* | 0.000 | 0.000 | 0.000 | 0.000 | 0.002 | 0.000 | 0.000 | 0.000 | 0.000 | 0.000 | DQ799536 |
| OPU406 | 1 | *uncultured clostridiales* | 0.000 | 0.000 | 0.000 | 0.000 | 0.002 | 0.000 | 0.000 | 0.000 | 0.000 | 0.000 | HW066368 |
| OPU407 | 1 | *uncultured clostridiales* | 0.000 | 0.000 | 0.000 | 0.000 | 0.004 | 0.000 | 0.000 | 0.000 | 0.000 | 0.000 | EU506672 |
| OPU408 | 1 | *uncultured clostridiaceae* | 0.000 | 0.000 | 0.000 | 0.000 | 0.002 | 0.000 | 0.000 | 0.000 | 0.000 | 0.000 | DQ394594 |
| OPU409 | 9 | *Fusobacterium sp.* | 14.499 | 0.265 | 0.000 | 0.000 | 0.004 | 0.000 | 0.006 | 0.000 | 27.795 | 4.420 | HM037995 |
| OPU409 | 2 | *uncultured clostridiaceae* | 0.000 | 0.009 | 0.000 | 0.000 | 0.000 | 0.000 | 0.000 | 0.005 | 0.000 | 0.002 | HQ790981 |
| OPU410 | 2 | *Fusobacterium sp.* | 0.235 | 0.000 | 0.000 | 0.000 | 0.000 | 0.000 | 0.000 | 0.000 | 0.000 | 0.030 | X77850 |
| OPU411 | 13 | *uncultured fusobacteriales* | 0.000 | 0.000 | 0.000 | 0.560 | 7.333 | 41.092 | 44.833 | 0.000 | 0.121 | 11.330 | FJ470422 |
| OPU412 | 14 | *uncultured fusobacteriales* | 0.000 | 0.000 | 0.000 | 0.061 | 0.597 | 0.714 | 0.520 | 0.000 | 0.008 | 0.234 | FJ470422 |
| OPU413 | 1 | *Leptotrichia buccalis* | 0.000 | 0.000 | 0.000 | 0.000 | 0.000 | 0.000 | 0.000 | 0.000 | 0.012 | 0.001 | CP001685 |
| OPU414 | 1 | *uncultured fusobacteriales* | 0.000 | 0.000 | 0.000 | 0.000 | 0.002 | 0.000 | 0.000 | 0.000 | 0.000 | 0.000 | FJ470422 |
| OPU415 | 1 | *Clostridium citroniae* | 0.060 | 0.000 | 0.000 | 0.000 | 0.000 | 0.000 | 0.000 | 0.000 | 0.000 | 0.008 | DQ279737 |
| OPU416 | 1 | *Clostridium aldenense* | 0.045 | 0.000 | 0.000 | 0.000 | 0.000 | 0.000 | 0.000 | 0.000 | 0.000 | 0.006 | DQ279736 |
| OPU417 | 1 | *Hungatella effluvii* | 0.084 | 0.000 | 0.000 | 0.000 | 0.000 | 0.000 | 0.000 | 0.000 | 0.000 | 0.011 | HE603919 |
| OPU418 | 1 | *Eubacterium uniforme* | 0.000 | 0.000 | 0.000 | 0.000 | 0.000 | 0.000 | 0.000 | 0.000 | 0.004 | 0.000 | GU269550 |
| OPU419 | 1 | *Eubacterium rectale* | 0.000 | 0.000 | 0.000 | 0.000 | 0.000 | 0.000 | 0.000 | 0.004 | 0.000 | 0.000 | L34627 |
| OPU420 | 2 | *Eubacterium cellulosolvens* | 0.000 | 0.000 | 0.000 | 0.000 | 0.000 | 0.005 | 0.000 | 0.000 | 0.000 | 0.001 | X71860 |
| OPU421 | 1 | *Clostridium sp.* | 0.000 | 0.000 | 0.000 | 0.000 | 0.003 | 0.000 | 0.000 | 0.000 | 0.000 | 0.000 | X76746 |
| OPU422 | 1 | *Cellulosilyticum lentocellum/C. ruminicola* | 0.000 | 0.000 | 0.000 | 0.008 | 0.000 | 0.000 | 0.000 | 0.000 | 0.000 | 0.001 | X71851 |
| OPU423 | 7 | *Peptoniphilus sp.* | 2.247 | 0.019 | 0.000 | 0.000 | 0.000 | 0.322 | 0.241 | 0.497 | 1.267 | 0.519 | GU440754/KF705042 |
| OPU424 | 1 | *uncultured clostridiales* | 0.000 | 0.007 | 0.000 | 0.000 | 0.000 | 0.000 | 0.000 | 0.000 | 0.000 | 0.001 | DQ353902 |
| OPU425 | 1 | *Phascolarctobacterium faecium* | 0.111 | 0.000 | 0.000 | 0.000 | 0.000 | 0.000 | 0.000 | 0.000 | 0.000 | 0.014 | X72865 |
| OPU426 | 1 | *Varibaculum cambriense* | 0.017 | 0.000 | 0.000 | 0.000 | 0.000 | 0.000 | 0.000 | 0.000 | 0.000 | 0.002 | AJ428402 |
| OPU427 | 1 | *Bifidobacterium bifidum* | 0.014 | 0.000 | 0.000 | 0.000 | 0.000 | 0.000 | 0.000 | 0.000 | 0.000 | 0.002 | U25952 |
| OPU428 | 1 | *Alloscardovia omnicolens* | 0.000 | 0.018 | 0.000 | 0.000 | 0.000 | 0.000 | 0.000 | 0.000 | 0.000 | 0.003 | AM419460 |
| OPU429 | 1 | *uncultured pasteurellaceae* | 0.000 | 0.000 | 0.000 | 0.000 | 0.042 | 0.000 | 0.000 | 0.000 | 0.000 | 0.005 | AF228001/X89379 |
| OPU430 | 4 | *Shewanella putrefaciens* | 0.000 | 0.110 | 0.000 | 0.007 | 0.000 | 0.000 | 0.000 | 0.060 | 0.023 | 0.023 | X81623 |
| OPU431 | 4 | *Halomonas elongata* | 0.000 | 0.413 | 0.000 | 0.000 | 0.036 | 0.000 | 0.000 | 0.174 | 0.047 | 0.081 | AM941743 |
| OPU432 | 5 | *Pelomonas saccharophila* | 0.000 | 0.113 | 0.000 | 0.009 | 0.017 | 0.000 | 0.000 | 0.042 | 0.016 | 0.024 | AB021407 |
| OPU433 | 1 | *Sphingomonas sp.* | 0.000 | 0.003 | 0.000 | 0.000 | 0.000 | 0.000 | 0.000 | 0.000 | 0.000 | 0.000 | HQ224549/JQ08327 |
| OPU434 | 1 | *uncultured phyllobacteriaceae* | 0.000 | 0.000 | 0.000 | 0.000 | 0.002 | 0.000 | 0.000 | 0.000 | 0.000 | 0.000 | HF565048 |
| OPU435 | 2 | *Brevundimonas sp.* | 0.000 | 0.005 | 0.000 | 0.000 | 0.000 | 0.000 | 0.000 | 0.000 | 0.005 | 0.001 | FR775448 |
| OPU436 | 1 | *uncultured bacteroidetes* | 0.000 | 0.007 | 0.000 | 0.000 | 0.000 | 0.000 | 0.000 | 0.000 | 0.000 | 0.001 | EU504660 |
| OPU437 | 1 | *Bacteroides uniformis* | 0.000 | 0.004 | 0.000 | 0.000 | 0.000 | 0.000 | 0.000 | 0.000 | 0.000 | 0.000 | AB050110 |
| OPU438 | 1 | *uncultured porphyromonadaceae* | 0.158 | 0.000 | 0.000 | 0.000 | 0.000 | 0.000 | 0.000 | 0.000 | 0.000 | 0.020 | AB078842/LM643867 |
| OPU439 | 1 | *Falsiporphyromonas endometrii* | 0.019 | 0.000 | 0.000 | 0.000 | 0.000 | 0.000 | 0.000 | 0.000 | 0.000 | 0.002 | HF969314 |
| Total | 98 |  | 18.161 | 0.977 | 0 | 0.646 | 8.060 | 42.133 | 45.600 | 0.786 | 29.366 | 16.844 |  |
