# Supplementary material for: Metataxonomics reveal vultures as a reservoir for Clostridium perfringens
Source: Emerg Microbes Infect. 2017 Feb 22;6(2):e9–. doi: 10.1038/emi.2016.137 (PMC5322324; doi:10.1038/emi.2016.137)
Supplement: Supplementary Table 7 [file emi2016137x11.docx]

**Supplementary Table S7 Primer sequences and diversity of *C. perfringens* MLST loci of each genes**

| Gene | Primer name | Primer sequence (5’-3’) | Amplicon size (bp) | Number of alleles |
| --- | --- | --- | --- | --- |
| *plc* | plc_F  plc_R | ATATGAATGGCAAAGAGGAAAC  AGTTTTTCCATCCTTTGTTTTG | 544 | 31 |
| *ddlA* | ddlA_F  ddlA_R | ATAATGGGGGATCATCAGTTGC  TTGTCATACCAGGTAATGTATTT | 393 | 28 |
| *dut* | dut_F  dut_R | TATATGCTAATAAGGATGTAT  CTGTAGTACCAAATCCACCACG | 381 | 25 |
| *glpK* | glpK_F  glpK_R | TGGGTTGAGCATGATCCAATGG  CACCTTTTGCTCCAAGGTTTGC | 574 | 27 |
| *gmk* | gmk_F  gmk_R | TAAGGGAACTATTTGTAAAGCC  TACTGCATCTTCTACATTATCG | 475 | 18 |
| *recA* | recA_F  recA_R | GCTATAGATGTTTTAGTTGTGG  CTCCATATGAGAACCAAGCTCC | 475 | 23 |
| *sod* | sod_F  sod_R | GATGCTTTAGAGCCATCAATAG  AATAATAAGCATGTTCCCAAAC | 478 | 30 |
| *tpiA* | tpiA_F  tpiA_R | AAATGTGAAGTTGTTGTTTGCC  CATTAGCTTGGTCTGAAGTAGC | 451 | 9 |
| *cpb*2 | CPB2_F  CPB2_R | CAAGCAATTGGGGGAGTTTA  GCAGAATCAGGATTTTGACCA | 200 |  |

The primer sequences of the eight MLST genes is according to Jost B. H. et al, 2006.

The primer sequences of *cpb*2 is according to Christoph G. et al., 2003
